# Attempted synthesis of a *meta*-metalated calix[4]arene

**DOI:** 10.3762/bjoc.15.195

**Published:** 2019-08-22

**Authors:** Christopher D Jurisch, Gareth E Arnott

**Affiliations:** 1 Department of Chemistry and Polymer Science, Stellenbosch University, Private Bag X1, Matieland, 7602, South Africa

**Keywords:** calixarene, inherent chirality, mesoionic carbene, mononitration, ruthenacycle

## Abstract

An evidence for the formation of a rare *meta*-metalated inherently chiral calix[4]arene is described. Our strategy involved using a mesoionic carbene to direct C–H activation, but proved to form an unexpectedly unstable intermediate that was identified through high-resolution mass spectrometry. On route to our target, a new optimized method to mononitrocalix[4]arenes was developed, including optimized and high yielding transformations to azide and 1,2,3-triazole derivatives which may have application in other areas of research.

## Introduction

**Figure 1:**
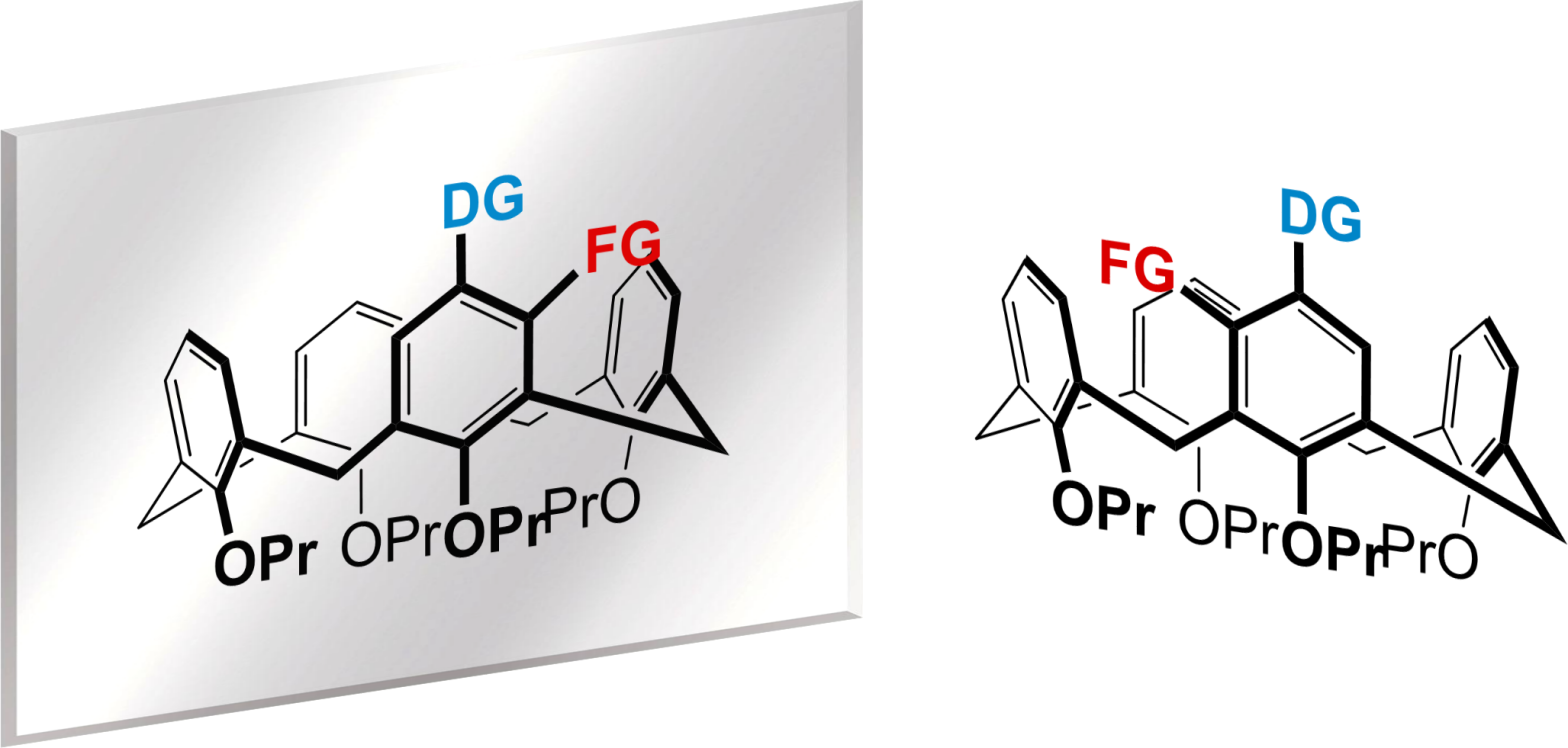
Inherent chirality generated by *meta*-substitution – the two structures are non-superposable mirror images, thus enantiomers. DG = directing group; FG = any functional group.

Calix[4]arenes are a class of diverse macrocyclic compounds which have been the subject of extensive study since the mid-twentieth century [1]. Obtained from the base-catalyzed condensation of *para-*alkylated phenols and formaldehyde, calix[4]arenes have cyclic bowl-like structures which provide various sites for controlled chemical modifications. The versatility of calix[4]arenes has resulted in applications in several fields, including, amongst others, the use as ligands in transition metal catalysis [2–3], in solid-phase gas sorption [4], as molecular sensors [5–6] and as biomimetic compounds [7–8]. Our own interest has been in the synthesis of inherently chiral calix[4]arenes whose chiral nature is a result of selective substitution on the calix[4]arene by various functional groups [9]. In particular, we have focused on the less-studied *meta*-substituted derivatives [10] whose inherent chirality is more commonly introduced by a suitable directing group [11–15] (see Figure 1), although two unusual methods have also been reported [16–17].

**Figure 2:**
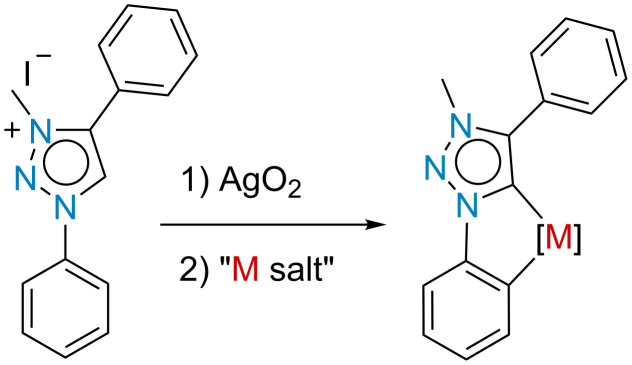
General approach by Albrecht for MIC directed cyclometalation via C–H activation; M = Ru(II), Ir(III) or Rh(III).

We have been looking at alternative methods for the *meta-*functionalization of calix[4]arenes, with the idea of C–H activation via transition metals appearing an attractive option. Lhoták and co-workers have already attempted this using a pyridyl sulfoxide directing group and palladium as the metal, but in this case the palladium caused a double C–H activation and formed an impressive bridge to the adjacent calix[4]arene aromatic ring [14]. The question for us was whether this bridge formation was a general principle or whether stable *meta-*metalated calix[4]arenes could be formed, isolated and used. For this, our attention turned to the work of Albrecht and co-workers on mesoionic carbenes (MICs) [18–22], particularly the fact that 1,4-diphenyl-substituted 1,2,3-triazol-5-ylideneruthenium(II), iridium(III) and rhodium(III) complexes underwent spontaneous, chemoselective cyclometalation via C–H bond activation of the N-bound phenyl group (see Figure 2) [19].

**Figure 3:**
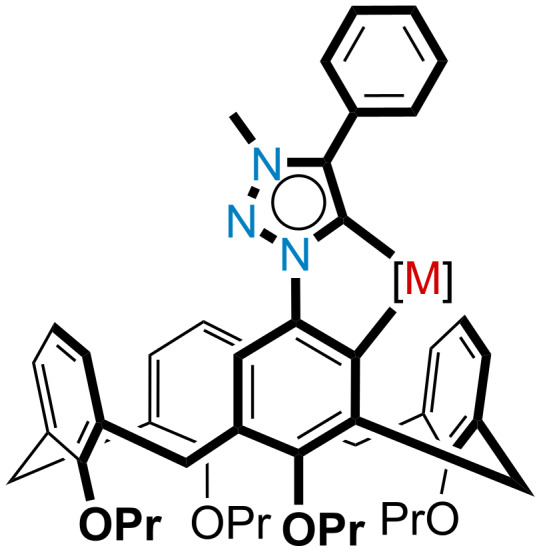
Concept of cyclometalated calix[4]arene target.

Incorporation of a mesoionic carbene directing group onto the upper rim of a calix[4]arene therefore offered potential for a new route to calix[4]arene C–H activation. Spontaneous cyclometalation of the MIC-functionalized calix[4]arene with a transition metal would provide access to a new type of *meta*-functionalized inherently chiral calix[4]arene (see Figure 3). Successful isolation of the mesoionic calix[4]arene metallocycle could have potential implications for asymmetric catalysis, due to the inherent chirality of the calix[4]arene, coupled with the point chirality from, for example, a tetrahedral ruthenium center. We therefore decided to apply the methods reported by Albrecht to a calix[4]arene system in order to prove this theory.

## Results and Discussion

### Model study

**Scheme 1:**
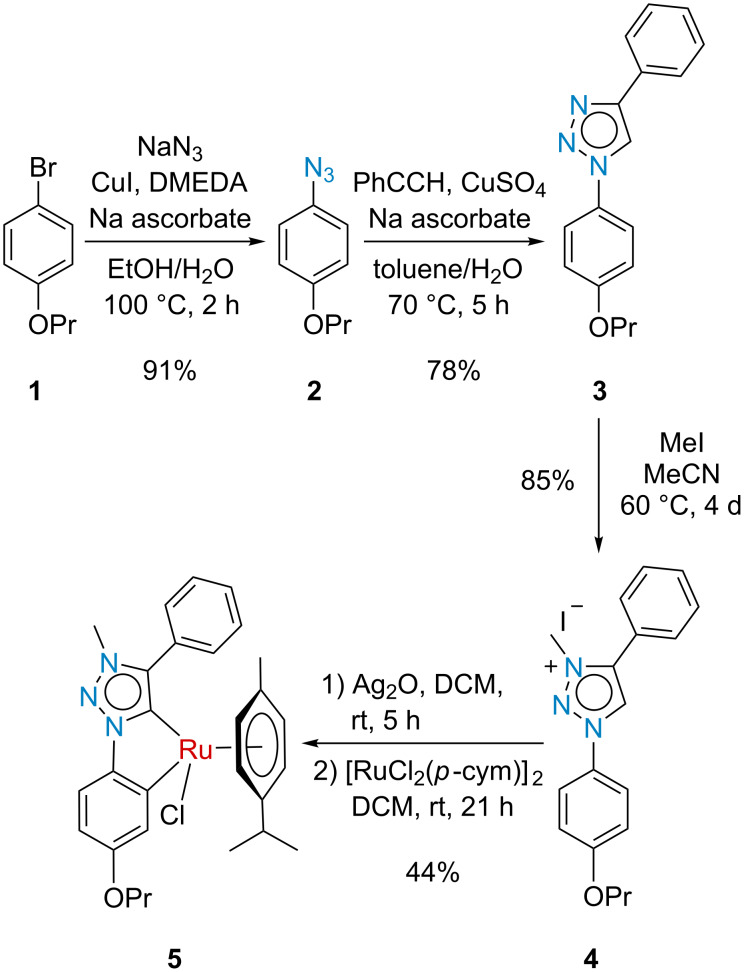
Synthesis of model mesoionic carbene **5**.

An unreported model ruthenium metallocycle was first synthesized, based on the work published by Albrecht and co-workers, in order to gain a better understanding of the metallocycle synthetic challenges. To this end, known 1-bromo-4-propoxybenzene (**1**) underwent an Ullmann-type coupling to give aryl azide **2**, which readily reacted with phenylacetylene in a copper-catalyzed Huisgen 1,3-dipolar cycloaddition to give 1,2,3-triazole **3** (Scheme 1). The formation of the ruthenacycle was then achieved using Albrecht’s method involving regioselective methylation of triazole **3**, followed by metalation of the triazolium salt **4** with silver oxide, which was immediately transmetalated with [RuCl_2_(*p*-cymene)]_2_. In our hands, purification using Albrecht’s trituration method was unsuccessful, however, neutral alumina column chromatography produced the desired model ruthenium metallocycle **5** in 44% yield which was comparable to the yields reported by Albrecht [20].

### Synthesis of the calix[4]arene ruthenacycle

**Scheme 2:**
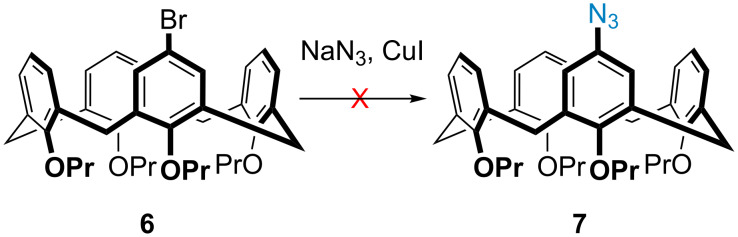
Attempted Ullmann-coupling to give monoazide **7**.

Satisfied that the published methods worked in our hands, we then set about synthesizing the targeted mesoionic calix[4]arene ruthenacycle. Our first key intermediate was monoazidocalix[4]arene **7**, whose synthesis was curiously more challenging than anticipated. Monobromocalix[4]arene **6** was first synthesized as the Ullmann-type coupling precursor in four steps using well established literature procedures [19,23–26]. Unfortunately, the Ullmann-type coupling route used in the model study was ultimately unsuccessful, despite many attempts using different reaction conditions. Our biggest challenge was deemed to be the poor solubility of the monobromocalix[4]arene in the reaction’s solvents, but even solving the solubility issue resulted in mixtures of products that were impossible to isolate to full purity (see Scheme 2 and Supporting Information File 1 for details). It should be noted that a similar reaction has been recently reported by Lagrost and Jabin [27], on a monobromocalix[4]arene, but in their case the pendant chains were ester groups which presumably helped with the solubility.

An alternative route involving an azide-Sandmeyer reaction on monoaminocalix[4]arene was then envisaged, since the necessary monoaminocalix[4]arene would be accessible via a previously reported mononitration method [28–31]. However, this method for mononitration of tetrapropoxycalix[4]arene **8**, which used a combination of nitric acid and acetic acid, proved inconsistent in our hands (see Supporting Information File 1 for more details). We therefore conducted a careful study to optimize the reaction using a more traditional combination of nitric acid and sulfuric acid (Table 1). A solution of tetrapropoxycalix[4]arene **8** in DCM was stirred at 0 °C and treated with nitric acid (70%) and concentrated sulfuric acid; after 300 min the solution had reached 18 °C, at which point the reaction was diluted with water and extracted into methylene chloride. Purification via silica gel flash column chromatography afforded mononitrocalix[4]arene **9** in 34% yield (Table 1, entry 1) with slightly less than 50% of the starting material remaining unreacted (overnitrated products were only obtained in trace amounts ≈9%). To reduce the reaction time, the reaction was repeated and the solution placed in a water bath at a constant temperature of 15 °C, after stirring at 0 °C for 5 minutes (Table 1, entry 2). After stirring for 145 minutes at 15 °C, mononitrocalix[4]arene **9** was obtained in 29% yield. As can be seen from entries 3–7, the reaction was repeated and the temperature of the bath increased to 20 °C. Upon increasing the reaction time from 50 minutes (Table 1, entry 3) to 80 minutes (Table 1, entry 6), the yield of mononitrocalix[4]arene **9** was increased from 23% to 48% after chromatography. Allowing the reaction to stir longer (Table 1, entry 7) started to produce more overnitrated products that then complicated the purification without increasing the yield. In our hands, this new method was found to be reproducible (gram scale) and was thus used to generate mononitrocalix[4]arene **9** for use in subsequent steps.

**Table 1:** Optimization results for the mononitration of calix[4]arene **9**.

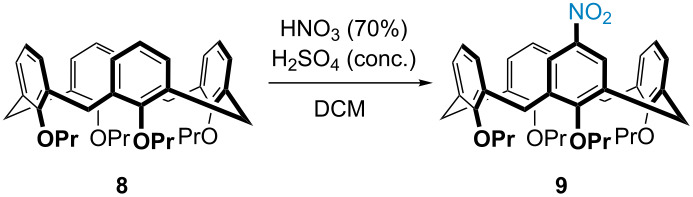			

Entry	Temp (°C)	Time (min)	Yield (%)

**1**	0 to 18	300	34
**2**	0 then 15	145	29
**3**	0 then 20	50	23
**4**	0 then 20	65	28
**5**	0 then 20	72	29
**6**	0 then 20	80	48
**7**	0 then 20	85	46

**Scheme 3:**
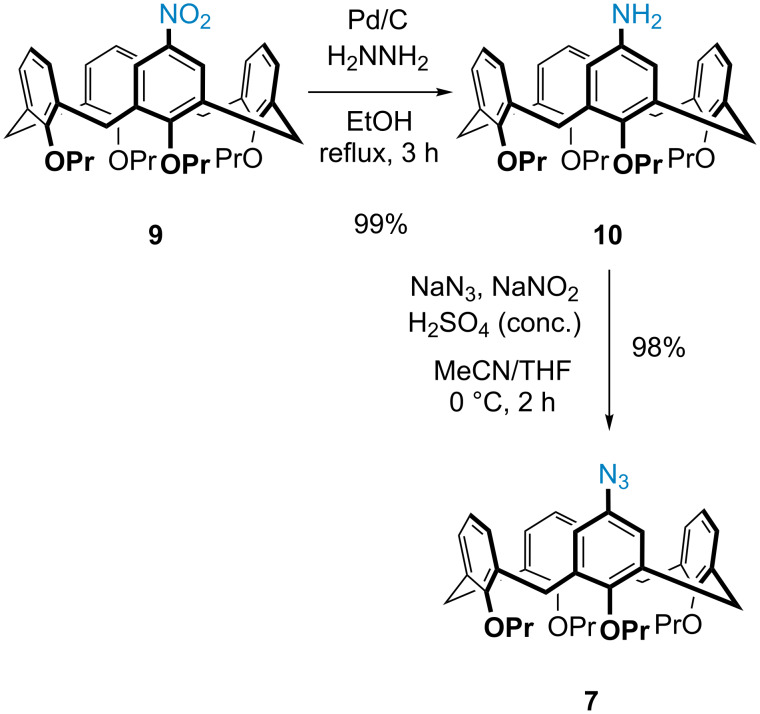
Synthesis of monoazidocalix[4]arene **7** under optimized conditions.

Reduction of mononitrocalix[4]arene **9** under transfer hydrogenation conditions (catalytic palladium on carbon and hydrazine hydrate) was found to be the most efficient method for obtaining monoaminocalix[4]arene **10** in essentially quantitative yields (Scheme 3). The azide-Sandmeyer reaction on monoaminocalix[4]arene **10** was then attempted using sodium nitrite and *p*-toluenesulfonic acid, but it became clear that the typical solvent conditions of water or methanol were not appropriate since monoaminocalix[4]arene **10** was insoluble in these. Changing the solvent to acetonitrile resulted in a cleaner reaction, although the yields were only around 40%, with starting material **10** remaining. Increasing the equivalents of the reagents and concentration of the reaction did little to improve the yield, so further optimization was needed. After numerous attempts, it was found that a 1:1 ratio of CH_3_CN/THF was the optimal solvent system, and the nitrosonium intermediate should be generated separately, using sulfuric acid and sodium nitrite, prior to being added to the monoaminocalix[4]arene **10** at 0 °C. After diazotization (15 min), an aqueous solution of sodium azide was added dropwise. After two hours at 0 °C the reaction was extracted with ethyl acetate, and the monoazidocalix[4]arene **7** obtained in 98% yield (Scheme 3). At this point it should be noted that our route to the monoazide is comparable to the similar route to a monoazidocalix[4]arene reported by Lagrost and Jabin [27]. Both of the routes involve four steps from calix[4]arene with overall yields of 40%, although in our case column chromatography was only used once (to purify the monoazidocalix[4]arene **7**), whilst the other procedure needed chromatography at each step.

**Scheme 4:**
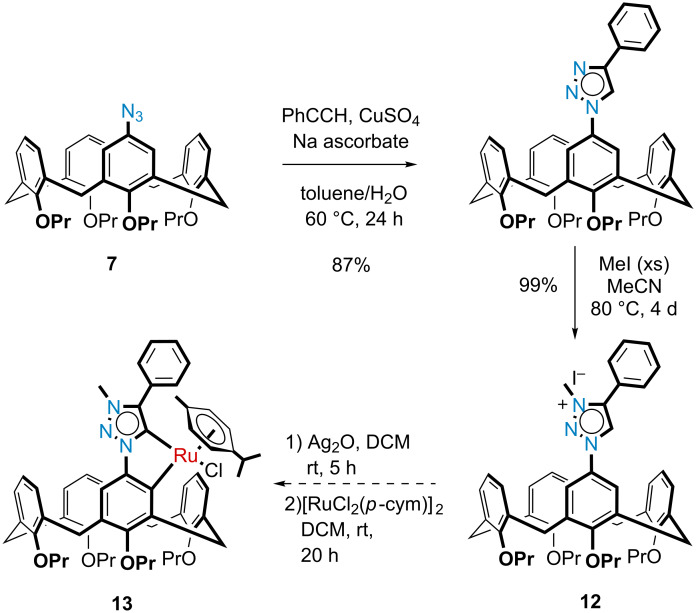
Synthesis of the putative calix[4]arene mesoionic carbene ruthenium complex **13**.

Introduction of the triazole moiety onto the upper rim of the calix[4]arene was then achieved with a copper-catalyzed Huisgen 1,3-dipolar cycloaddition between monoazidocalix[4]arene **7** and phenylacetylene, affording monotriazolocalix[4]arene **11** in 87% yield (Scheme 4). Methylation of the triazole was also achieved using excess iodomethane in a rather protracted reaction, to yield the calix[4]arene triazolium iodide salt **12** in effectively quantitative yield.

**Figure 4:**
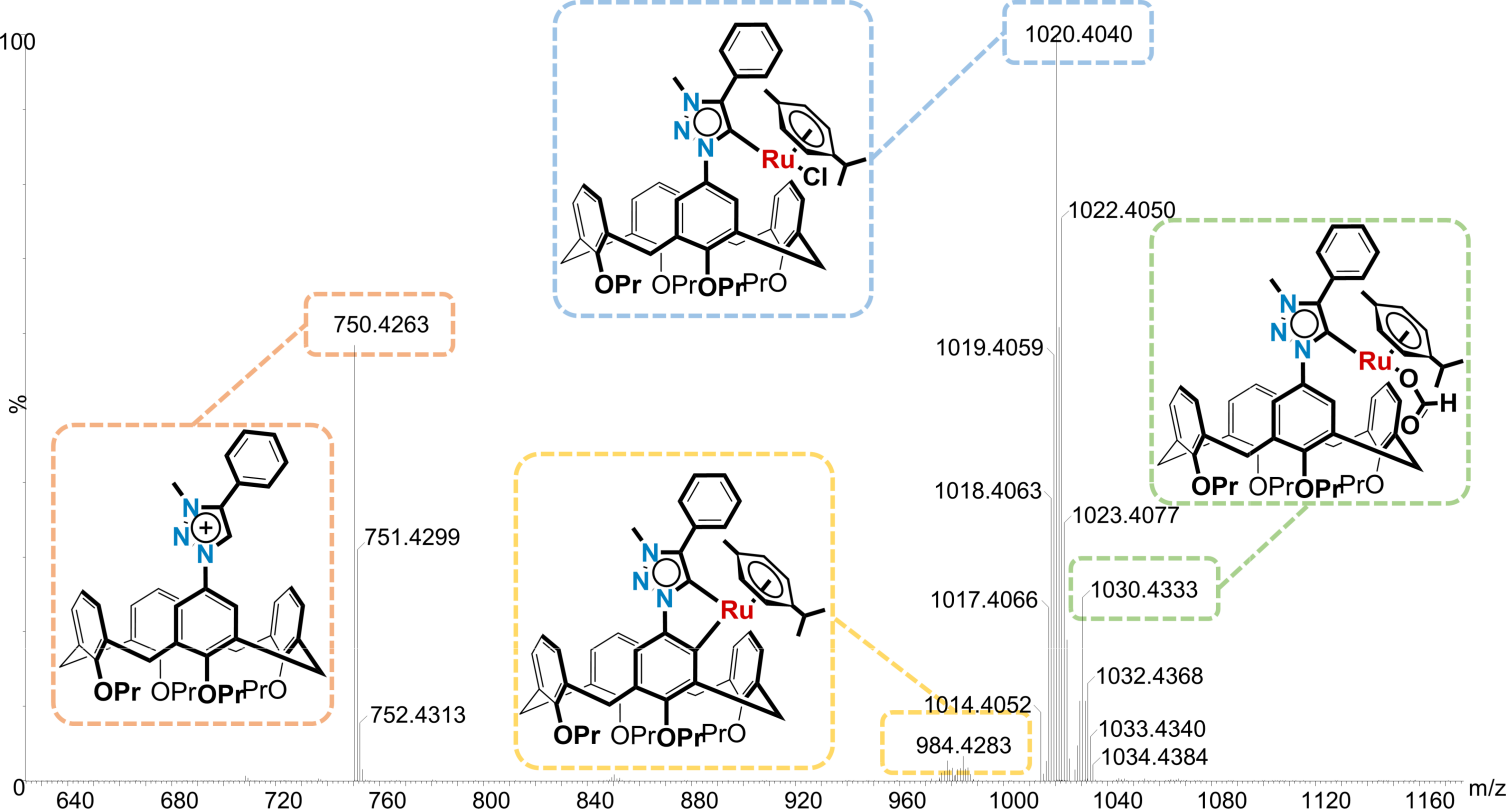
High-resolution mass spectrum (ESI^+^) of putative ruthenacycle calix[4]arene **13**.

In the final step towards a mesoionic calix[4]arene metallocycle, the synthesis was unfortunately not as trivial or as successful as we had hoped. The metalation reaction, using the methods described by Albrecht and co-workers [19], were repeated a number of times. In all cases the problem appeared that the product was unstable, which was unexpected since Albrecht has reported that their complexes were stable for months in both solution and the solid state. Even our own model study showed that the complex was easy to work with and manipulate, but we needed to concede that the calix[4]arene may create a far too sterically congested environment. Practically, we could observe the successful formation of the silver(I) intermediate via TLC analysis, and then also the putative ruthenacycle as well. After following Albrecht’s trituration method for purification, the ^1^H NMR spectrum revealed the loss of the triazole methine signal at δ 8.92 ppm, but was not clean enough to confirm the desired product. One complicating factor was that the ruthenium center is itself chiral and so the desired product would be expected to appear as a mixture of diastereomers since two new chiral centers would be formed. However, the ^1^H NMR spectroscopic evidence for this was speculative at best and would suggest a very small quantity of the desired product (see Supporting Information File 1, Figure S34). Another main contaminant was the dichloro(*p*-cymene)ruthenium(II) dimer, which we could not eliminate even after using sub-stoichiometric quantities in the reaction. Attempted purification via alumina flash chromatography returned material that was assigned as the triazolium chloride salt (i.e., Cl^–^ salt of triazolium **12**; based on chemical shifts in the ^1^H and ^13^C NMR that were consistent with a literature report [32]). This was presumably due to ruthenium undergoing a reductive elimination and the chloride remaining on the triazolium moiety. Crystallization also proved to be futile after many attempts under many different conditions. The only crystals that formed were either found to be the dichloro(*p*-cymene)ruthenium(II) dimer or the triazolium chloride salt already observed. As a last attempt to find evidence for the ruthenacycle, a high-resolution mass spectrum was acquired directly after the reaction was completed. The most important isotopic distribution detected was at 984.4283 Da, which corresponded to the expected [M − Cl]^+^ ion with Δ*m*_i_ = 2.30 ppm (Figure 4). However, this peak was very small and overlapped slightly with another isotopic distribution pattern that corresponded to [M + H − C_3_H_6_], i.e., loss of propene (see Supporting Information File 1 for a closer view of this). The major peak observed had a monoisotopic mass of 1020.4040 Da which fitted the predicted value for [M + H]^+^ of 1020.4020 Da (Δ*m*_i_ = 1.95 ppm) including the isotopic distribution pattern (Figure 4). Unfortunately, whilst this could represent the ruthenacycle, the more likely assignment would be for the ruthenium simply complexed to the mesoionic carbene (shown in Figure 4). Support for this comes from the peak at 750.4263 Da, which corresponded to the [triazolium **12** − halogen]^+^ ion, which was unlikely due to remaining starting material since this was not detected by NMR spectroscopy. Instead, this was likely the result of the molecular ion losing the ruthenium and subsequently protonated in the mass spectrometer; this triazolium signal was also not seen in our model study. A final peak at 1030.4333 Da was assigned as [M – Cl + formic acid]^+^ (formic acid was present in the injection media), which also shed no light on whether this was the open or closed form of the ruthenium complex. Nevertheless, we were confident that the ruthenacycle had been formed, but only in a very small amount.

We therefore considered that the ruthenacycle formation might be severely retarded by steric encumbrance of the calix[4]arene bowl. In this case, leaving the reaction for an extended time might prove a higher yield of the ruthenacycle. However, repeating the procedure at 30 °C for 5 days, or 2 weeks at room temperature (ca. 25 °C) returned a mixture whose analysis was identical in all respects to the previous experiments. This was an unexpected result and suggests that the C–H activation process is reversible, driven by the steric demand of the calix[4]arene.

## Conclusion

Whilst our inability to isolate and purify the calix[4]arene ruthenacycle **13** for full characterization was frustrating, the evidence did suggest that the reaction had worked, albeit in a very low yield. The major mass spectrum peak might be interpreted as successful formation of the ruthenacycle, but we believe that this is not the case and that the ruthenium complex shown in Figure 4 is the more likely interpretation. What was also important to note was that the reaction did not result in the oxidative bridge formation reported by Lhoták when they attempted a *meta*-functionalization of a calix[4]arene with palladium. The NMR and mass spectra were proof of this. We have also developed a more robust mononitration method applicable to calix[4]arenes and an optimized method to azide/triazole-functionalized calix[4]arenes which may be useful in other applications.

## Supporting Information

File 1 Experimental details and spectral data for all compounds (including failed reactions).
