# Supplementary material for: Attempted synthesis of a meta-metalated calix[4]arene
Source: Beilstein J Org Chem. 2019 Aug 22;15:1996–2002. doi: 10.3762/bjoc.15.195 (PMC6719732; doi:10.3762/bjoc.15.195)
Supplement: File 1 — Experimental details and spectral data for all compounds (including failed reactions). [file Beilstein_J_Org_Chem-15-1996-s001.pdf]

BEILSTEIN JOURNAL OF ORGANIC CHEMISTRY

Supporting Information

for

Attempted synthesis of a *meta*-metalated calix[4]arene

Christopher D. Jurisch and Gareth E. Arnott

Beilstein J. Org. Chem. **2019,** *15,* 1996–2002. doi:10.3762/bjoc.15.195

Experimental details and spectral data for all compounds (including failed reactions)

License and Terms: This is a supporting information file under the terms of the Creative Commons Attribution License (http://creativecommons.org/licenses/by/4.0). Please note that the reuse, redistribution and reproduction in particular requires that the authors and source are credited.
The license is subject to the *Beilstein Journal of Organic Chemistry* terms and conditions: (https://www.beilstein-journals.org/bjoc)

General practices

Chemicals used in these experiments were purchased from Merck or Sigma-Aldrich. Tetrahydrofuran and toluene were distilled under nitrogen from sodium wire/sand using benzophenone as an indicator. Dichloromethane and acetonitrile were distilled under nitrogen from calcium hydride. Other reagents requiring purification were purified according to standard procedures.[1]

For inert syntheses, glassware was oven dried. Thereafter it was placed under vacuum of <0.5 mmHg and cyclically flushed with argon and evacuated until it had reached room temperature. All reactions were performed under a positive pressure of 2.8 kPa of 5.0 grade argon (Air Products). Low temperature reactions were performed in a Dewar containing solid CO_2 in acetone (−78 °C) or ice in water (0 °C).

^{1}H NMR and ^{13}C NMR spectra were obtained using Varian 300 MHz VNMRS, Varian 400 MHz Unity INOVA and Varian 600 MHz Unity INOVA NMR instruments. Chemical shifts (δ) were recorded using the residual chloroform-*d* peaks (δ 7.26 ppm for ^{1}H NMR and δ 77.16 ppm for ^{13}C NMR). All chemical shifts are reported in ppm and all spectra were obtained at 25 °C. IR spectra were obtained using a Thermo Nicolet Nexus FTIR instrument using the ATR attachment. Mass spectra were collected using positive ESI on a Waters SYNAPT G2 QTOF mass spectrometer by CAF (Central Analytical Facility) at Stellenbosch University. Melting points were obtained using a Gallenkamp Melting Point Apparatus.

Column chromatography was performed using 230–400 nm silica gel or neutral alumina, and thin layer chromatography was performed using Macherey-Nagel DC-Fertigfolien ALUGRAM Xtra SIL G/UV_{254} or Alox N/UV_{254} TLC plates. Petroleum ether, ethyl acetate, dichloromethane and/or methanol were used individually or in combination as solvents for all chromatography. Compounds were visualised on TLC using UV light (254 nm) and 10% PPh_3 in DCM, as well as ninhydrin (NIN) stains.

1-Bromo-4-propoxybenzene,[2] [(η^6-*p*-cymene)$RuCl_2$]$_2$,[3] and tetrapropoxycalix[4]arene[4] were prepared according to literature procedures.

Compound characterisations

1-Azido-4-propoxybenzene (2)

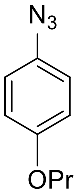

A two-necked, argon-purged round-bottom flask (50 mL) with a tap was charged with 1-bromo-4-propoxybenzene (215 mg, 1.00 mmol) and 6.0 mL EtOH/H_2O (7:3 (v/v)). The solvent was degassed three times using the freeze-pump-thaw method (FPT) and the flask placed under an inert argon atmosphere. Sodium azide (130 mg, 2.00 mmol, 2 equiv), sodium ascorbate (10 mg, 0.05 mmol, 0.05 equiv), CuI (19 mg, 0.10 mmol, 0.1 equiv) and *N*,*N*'-dimethylethylenediamine (DMEDA) (16 µL, 0.15 mmol, 0.15 equiv) were added to the flask, and the resulting blue suspension was degassed one more time using FPT. The reaction mixture was heated under reflux at 100 °C for 2 h until TLC showed complete consumption of the starting material. The dark blue suspension was cooled to room temperature and was then quenched with 1 M HCl (5 mL). The mixture was diluted with EtOAc (10 mL), the layers separated, and the organic phase washed with 1 M HCl (5 mL) and saturated $NaHCO_3$ (2 × 10 mL). The organic phase was dried over $MgSO_4$ and the solvent removed under reduced pressure to yield a dark brown viscous oil. Purification was achieved using silica gel flash column chromatography (EtOAc/PET 3:97), which yielded azide **2** as a dark red oil (162 mg, 0.91 mmol, 91%).

R

IR (ATR, cm^{-1}): 2965 (C-H), 2107 (N=N=N), 1501 and 1473 (C=C), 1239 and 978 (C-O), 822 (C-H)

^{1}H NMR (400 MHz, CHLOROFORM-*d*) δ ppm 6.95 (d, 2H, $^3J_{HH}$ = 9.1 Hz, Ar*H*), 6.88 (d, 2H, $^3J_{HH}$ = 9.1 Hz, Ar*H*), 3.90 (t, 2H, $^3J_{HH}$ = 6.6 Hz, OC*H*$_2$CH$_2$), 1.85 – 1.76 (m,* 2H, CH$_2$C*H*$_2$CH$_3$), 1.04 (t, 3H, $^3J_{HH}$ = 7.3 Hz, CH$_2$CH$_2$C*H*$_3$)

^{13}C{^{1}H} (400 MHz, CHLOROFORM-*d*) δ ppm 156.7, 132.3, 120.1, 115.9, 70.1, 22.7, 10.6.

Both HRMS–positive and HRMS–negative produced inconclusive results.

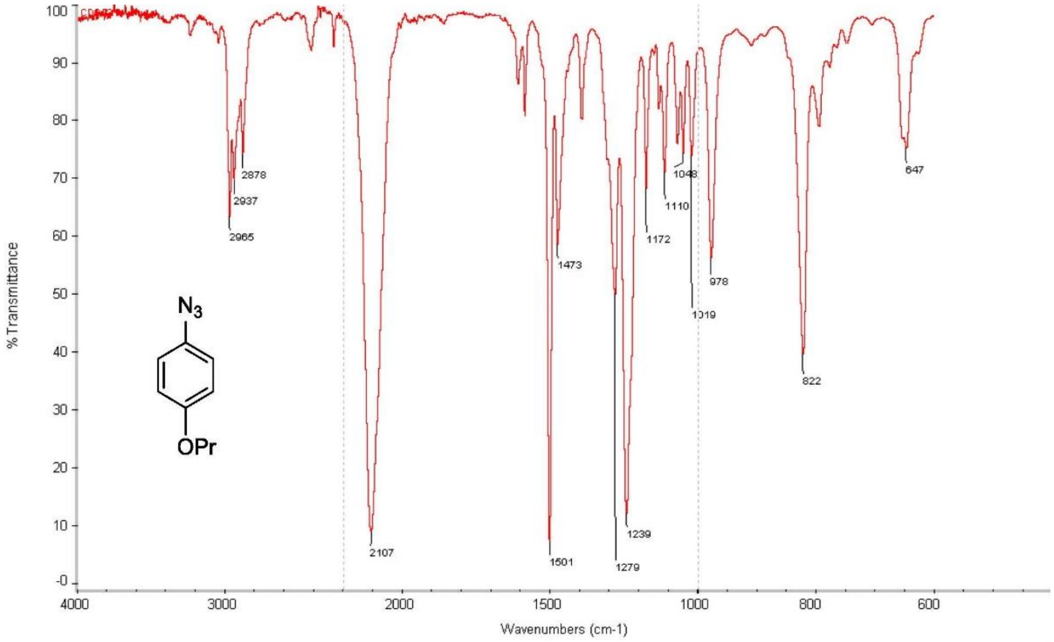

Figure S1: IR spectrum of 1-azido-4-propoxybenzene (**2**)

* The different *J*-coupling values experienced by these protons ($^3J_{HH}$ = 7.3 Hz, CH$_2$CH$_2$C*H*$_3$; $^3J_{HH}$ = 6.6 Hz, OC*H*$_2$CH$_2$) meant that this signal was expected to appear as a quartet of triplets. The resolution of the ^{1}H NMR spectrum did not allow for this, so a multiplet was observed. This was also the case for model compounds **3**, **4** and **5**.

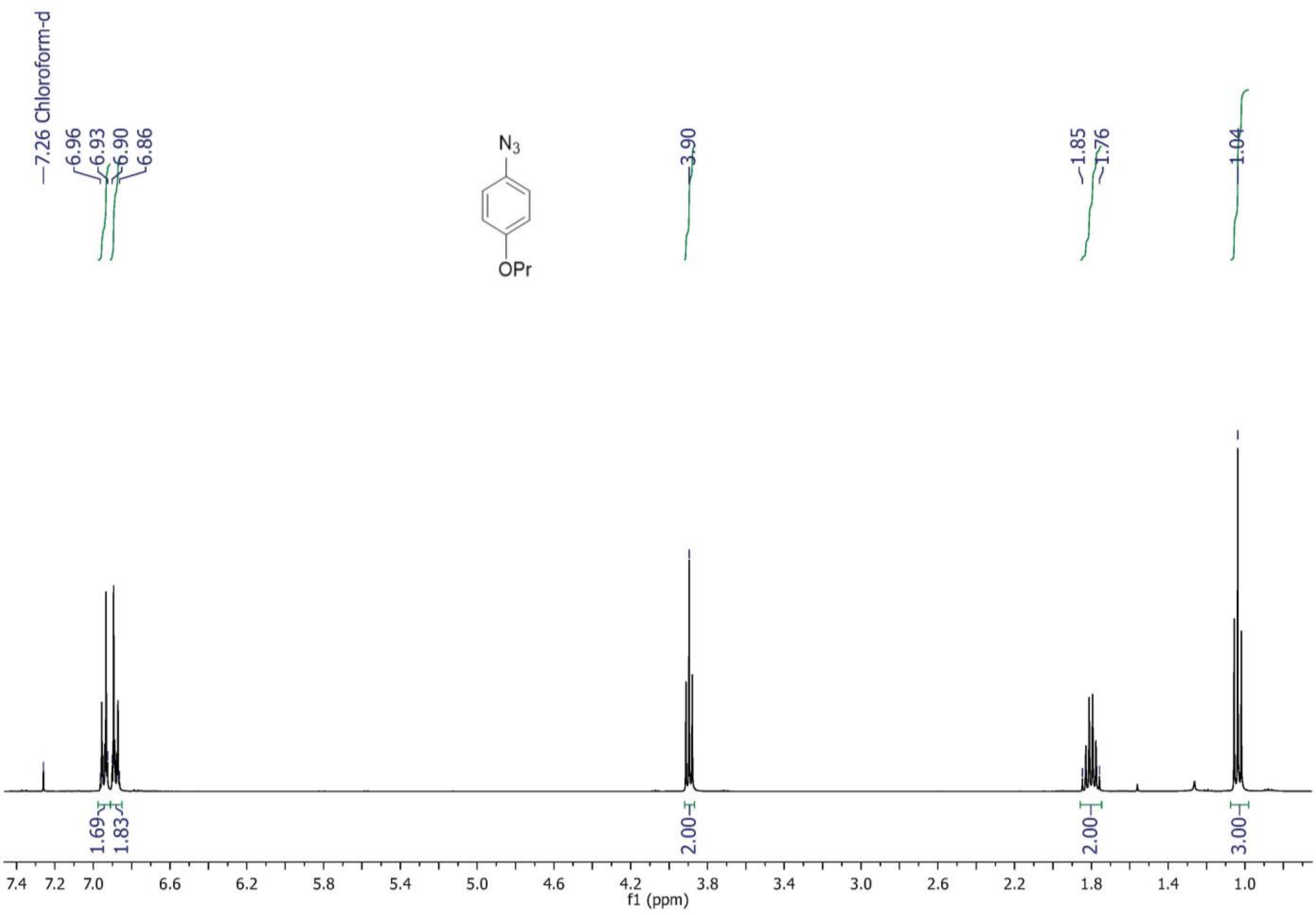

Figure S2: ^{1}H NMR spectrum of 1-azido-4-propoxybenzene (**2**)

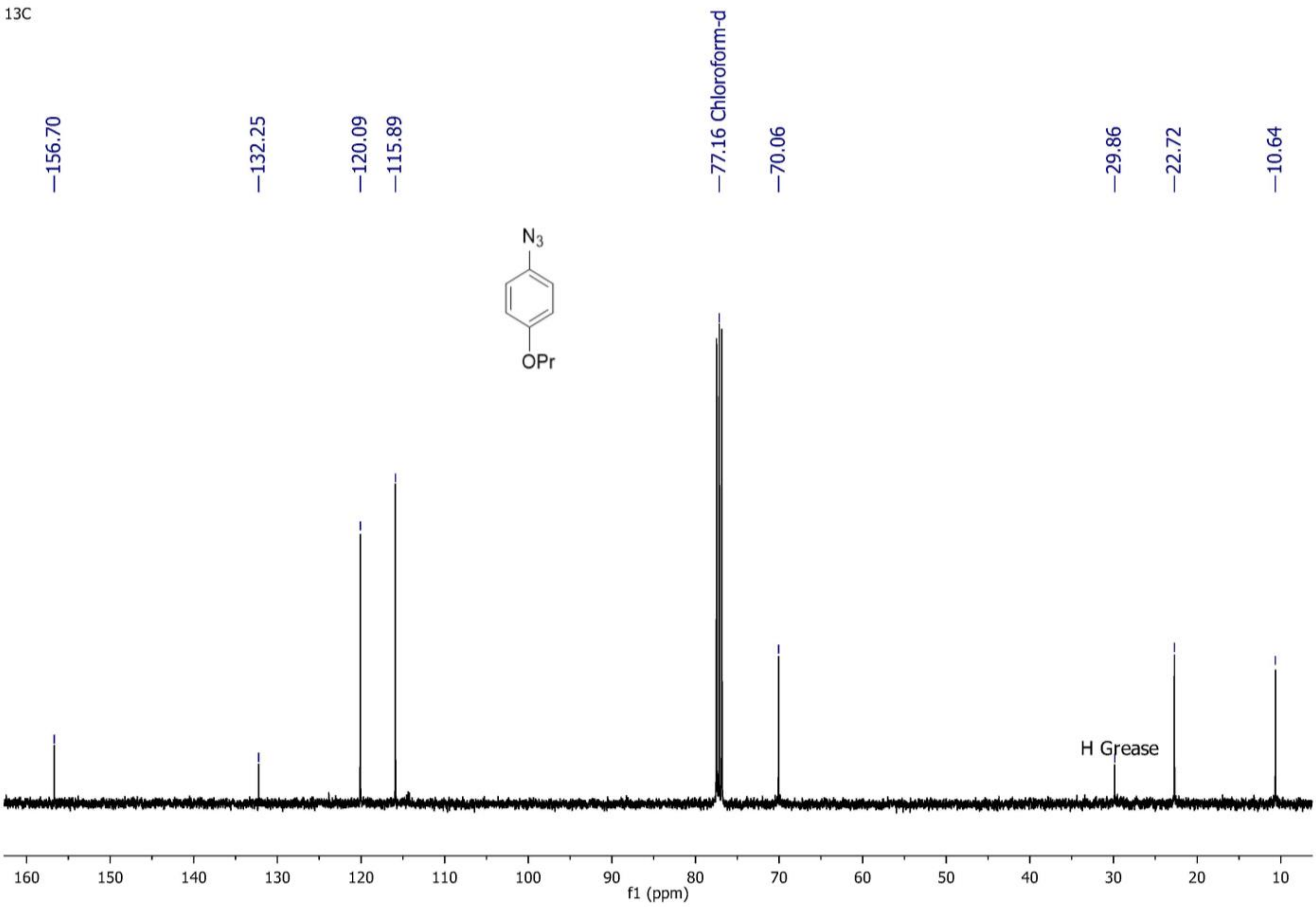

Figure S3: ^{13}C NMR spectrum of 1-azido-4-propoxybenzene (**2**)

4-Phenyl-1-(4-propoxyphenyl)-1*H*-1,2,3-triazole (3)

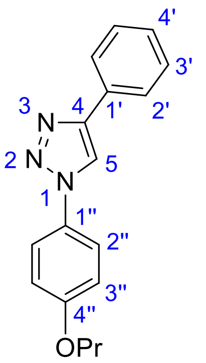

A solution of 1-azido-4-propoxybenzene (**2**, 200 mg, 1.13 mmol), phenylacetylene (136 µL, 1.24 mmol, 1.1 equiv), $CuSO_4{\cdot}5H_2O$ (20 mg, 80 µmol, 0.07 equiv) and sodium ascorbate (31 mg, 0.16 mmol, 0.14 equiv) in toluene (5.0 mL) and H_2O (1.0 mL) was placed under an inert atmosphere of argon in a sealed vial and stirred at 70 °C for 5 hours. TLC showed complete consumption of the starting material and the solution was cooled to rt. Saturated NH_4Cl (10 mL) was added and the product was extracted with EtOAc (2 × 20 mL). The two layers were separated and the combined organic layers were washed with saturated NH_4Cl (10 mL). The organic layer was separated and dried over $MgSO_4$, and the solvent removed under reduced pressure. Purification was achieved *via* silica gel flash column chromatography (EtOAc/PET 12:88), yielding triazole **3** as an off-white solid (246 mg, 0.880 mmol, 78%).

$\boldsymbol{R_f}$ = 0.30 (EtOAc:PET 12:88)

Mp = 140 – 142 °C

IR (ATR, cm^{-1}): 3054 (C-H), 1519 (C=C), 1265 and 1042 (C-O), 735 (C-H)

^{1}H NMR (400 MHz, CHLOROFORM-*d*) δ ppm 8.10 (s, 1H, H_5), 7.92 – 7.88 (2H, m, Ar$H_{2'}$), 7.67 (d, 2H, $^3J_{HH}$ = 9.0 Hz, Ar$H_{2''}$), 7.46 (dd [app. t], 2H, $^3J_{HH}$ = 7.3 Hz, Ar$H_{3'}$), 7.36 (tt, 1H, $^3J_{HH}$ = 7.3 Hz, $^4J_{HH}$ = 1.3 Hz, Ar$H_{4'}$), 7.03 (d, 2H, $^3J_{HH}$ = 9.0 Hz, Ar$H_{3''}$), 3.98 (t, 2H, $^3J_{HH}$ = 6.6 Hz, OCH_2CH_2), 1.91 – 1.79 (m, 2H, $CH_2CH_2CH_3$), 1.07 (t, 3H, $^3J_{HH}$ = 7.3 Hz, $CH_2CH_2CH_3$)

^{13}C{^{1}H} (400 MHz, CHLOROFORM-*d*) δ ppm 159.5, 148.3, 130.5, 130.4, 129.0, 128.4, 125.9, 122.2, 118.0, 115.4, 70.1, 22.6, 10.6

HRMS–Positive: m/z $[M+H]^+$ calcd. for $C_{17}H_{18}N_3O$: 280.1450; found: 280.1454.

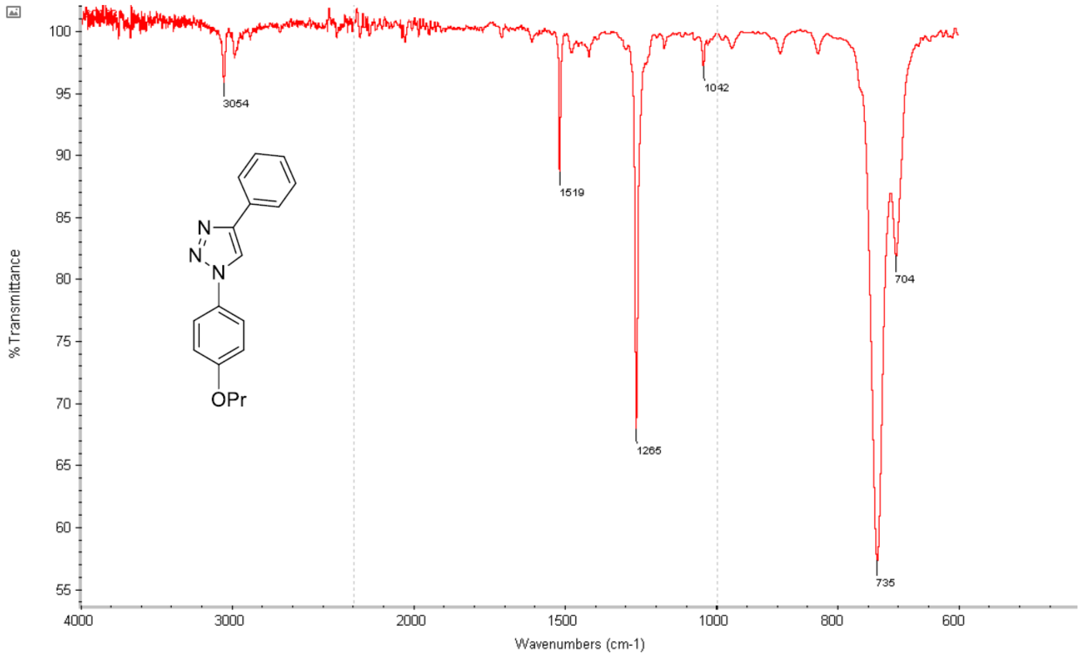

Figure S4: IR spectrum of 4-phenyl-1-(4-propoxyphenyl)-1*H*-1,2,3-triazole (**3**)

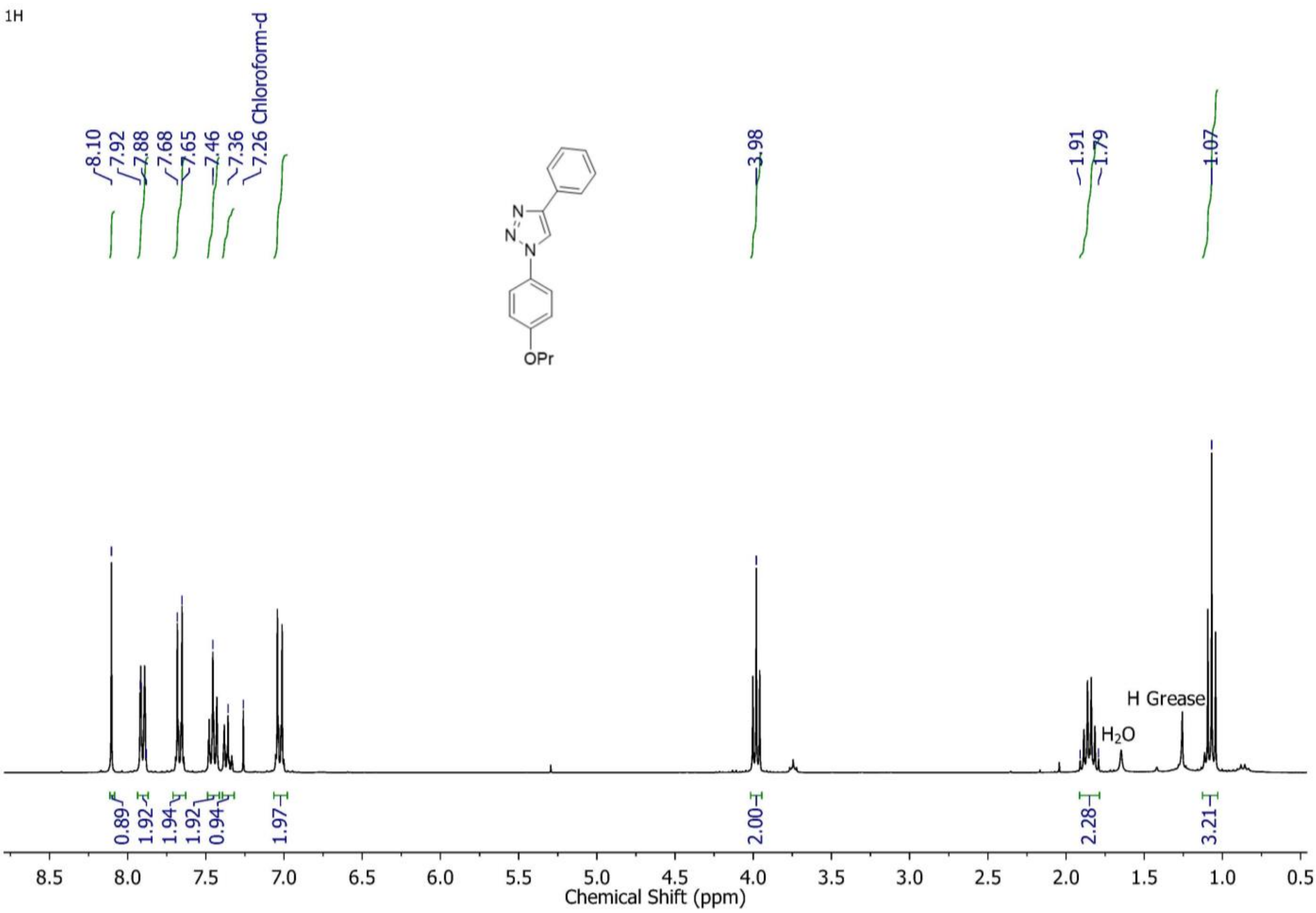

Figure S5: ^{1}H NMR spectrum of 4-phenyl-1-(4-propoxyphenyl)-1*H*-1,2,3-triazole (**3**)

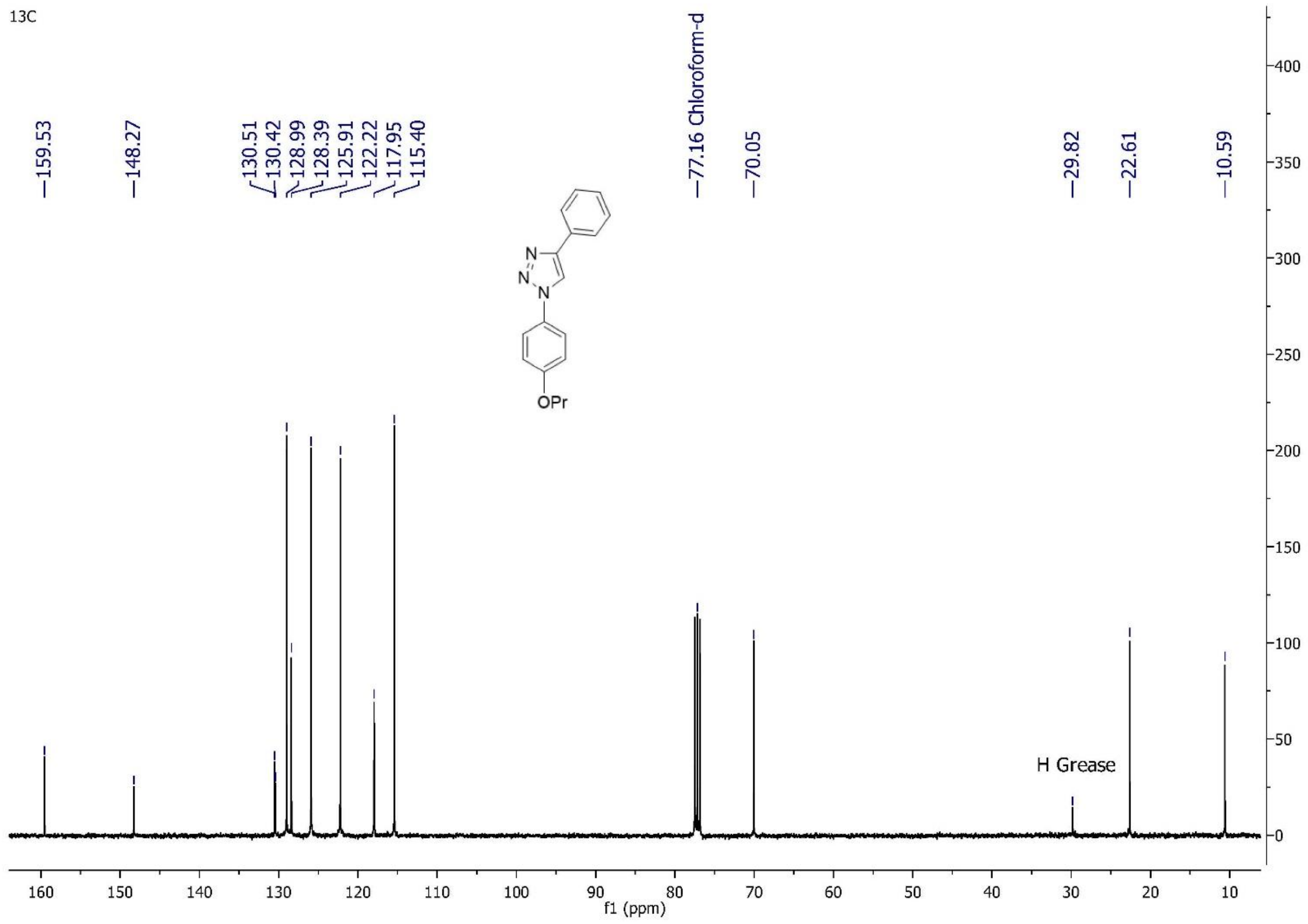

Figure S6: ^{13}C NMR spectrum of 4-Phenyl-1-(4-propoxyphenyl)-1*H*-1,2,3-triazole (**3**)

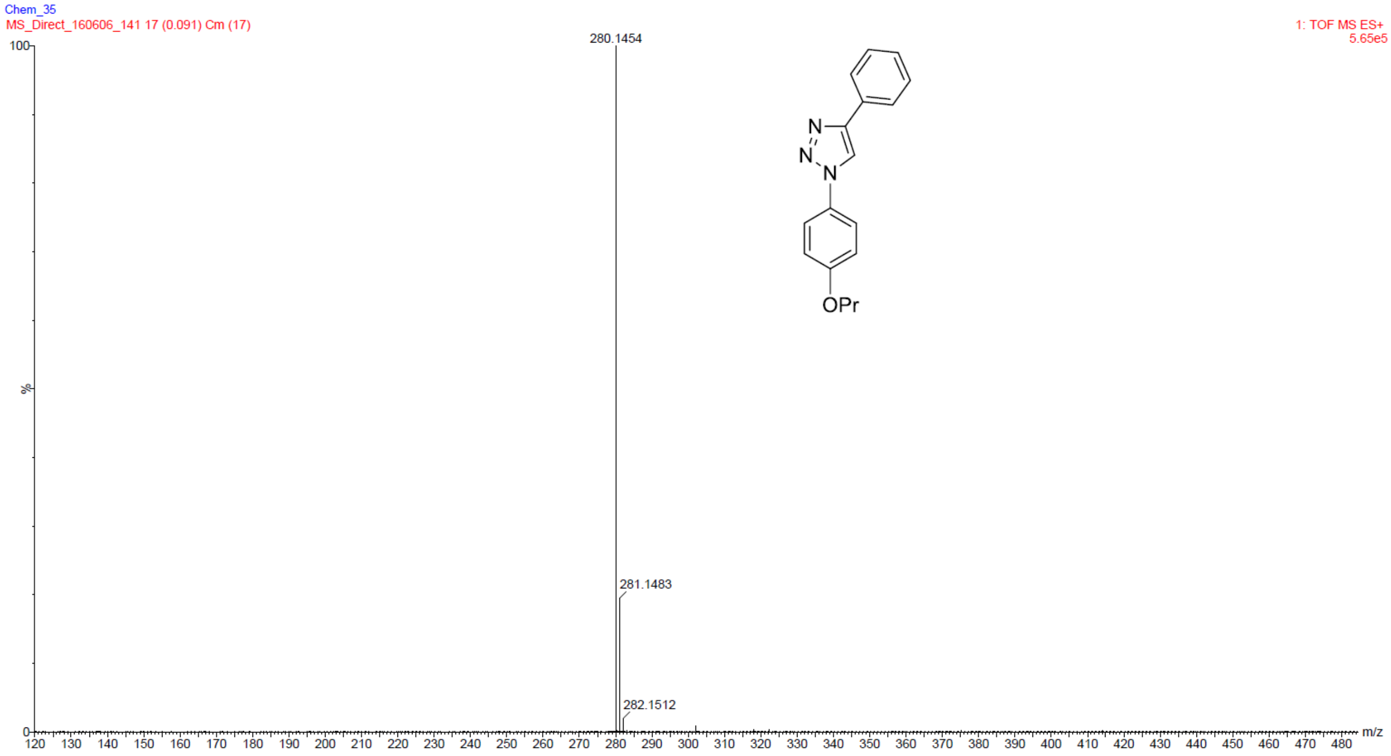

Figure 7: HRMS spectrum of 4-Phenyl-1-(4-propoxyphenyl)-1*H*-1,2,3-triazole (**3**)

3-Methyl-4-phenyl-1-(4-propoxyphenyl)-1*H*-1,2,3-triazolium iodide (4)

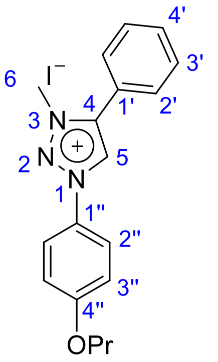

4-Phenyl-1-(4-propoxyphenyl)-1*H*-1,2,3-triazole (**3**, 250 mg, 0.895 mmol) was dissolved in MeCN (4.0 mL) in a sealable vial and MeI (366 µL, 5.88 mmol, 6.6 equiv) was added. The reaction was sealed under an inert atmosphere argon and stirred for 4 days at 60 °C, resulting in a clear orange solution. The solvent was removed in vacuo and the product was triturated using Et_2O/acetone. Compound **4** was obtained as an orange powder (322 mg, 0.764 mmol, 85%).

RR_f = 0.25 (MeOH:DCM 6:94)

Mp = 161 – 163 °C

IR (ATR, cm^{-1}): 3054 (C-H), 1264 (C-O), 733 (C-H)

^{1}H NMR (400 MHz, CHLOROFORM-*d*) δ ppm 9.61 (s, 1H, H_5), 8.07 (d, 2H, $^3J_{HH}$ = 9.0 Hz, Ar$H_{2''}$), 7.94 – 7.91 (m, 2H, Ar$H_{2'}$), 7.48 – 7.44 (m, 3H, Ar$H_{3'+4'}$), 6.98 (d, 2H, $^3J_{HH}$ = 9.0 Hz, Ar$H_{3''}$), 4.38 (s, 3H, H_6), 3.93 (t, 2H, $^3J_{HH}$ = 6.6 Hz, OCH_2CH$_2$), 1.87 – 1.75 (m, 2H, CH$_2$CH_2CH$_3$), 1.03 (t, 3H, $^3J_{HH}$ = 7.3 Hz, CH$_2$CH$_2$CH_3)

^{13}C{^{1}H} (400 MHz, CHLOROFORM-*d*) δ ppm 161.7, 143.9, 131.9, 130.1, 129.6, 127.5, 126.8, 123.4, 121.5, 115.8, 70.2, 40.0, 22.4, 10.5

HRMS–Positive: m/z [M–I]$^+$ calcd. for $C_{18}H_{20}N_3O$: 294.1606; found: 294.1600.

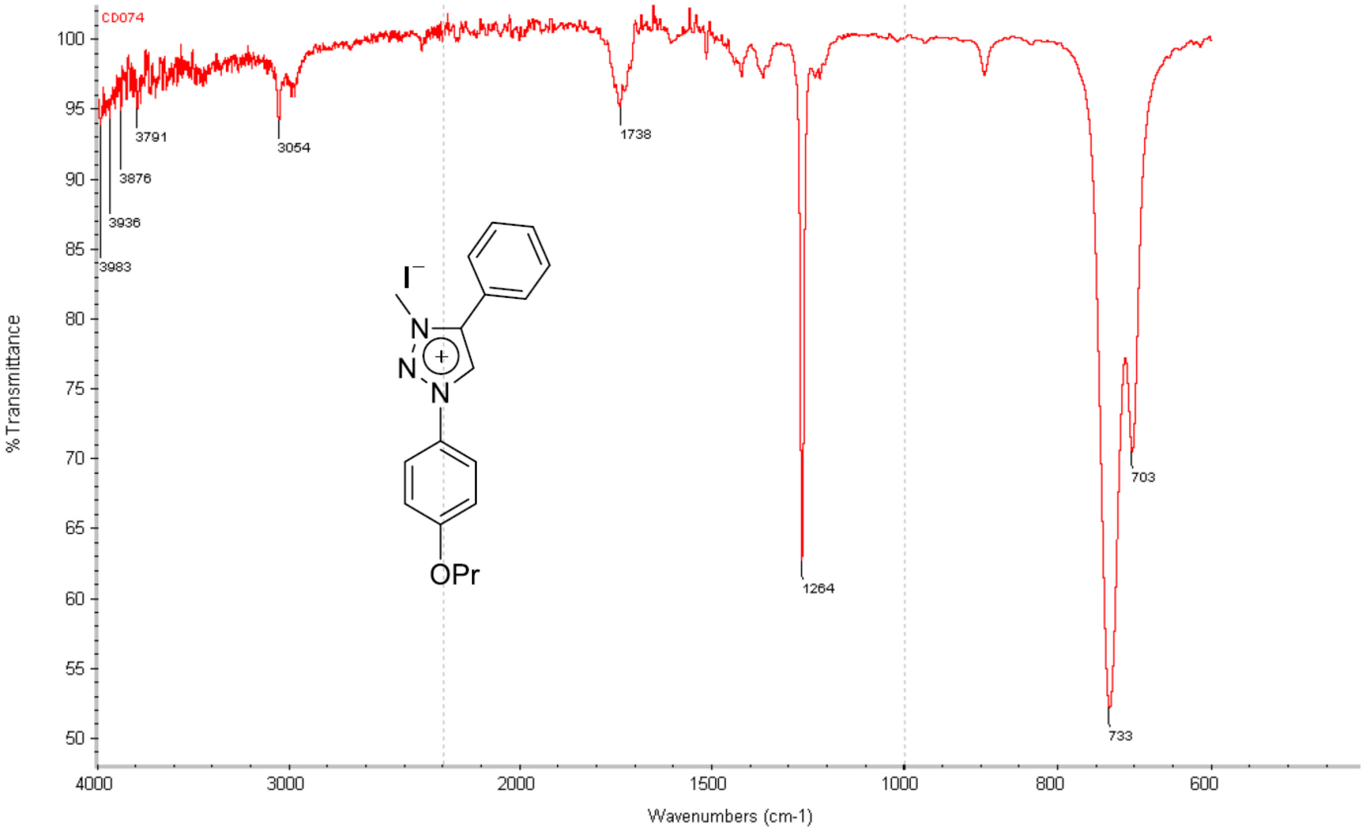

Figure S8: IR spectrum of 3-methyl-4-phenyl-1-(4-propoxyphenyl)-1*H*-1,2,3-triazolium iodide (**4**)

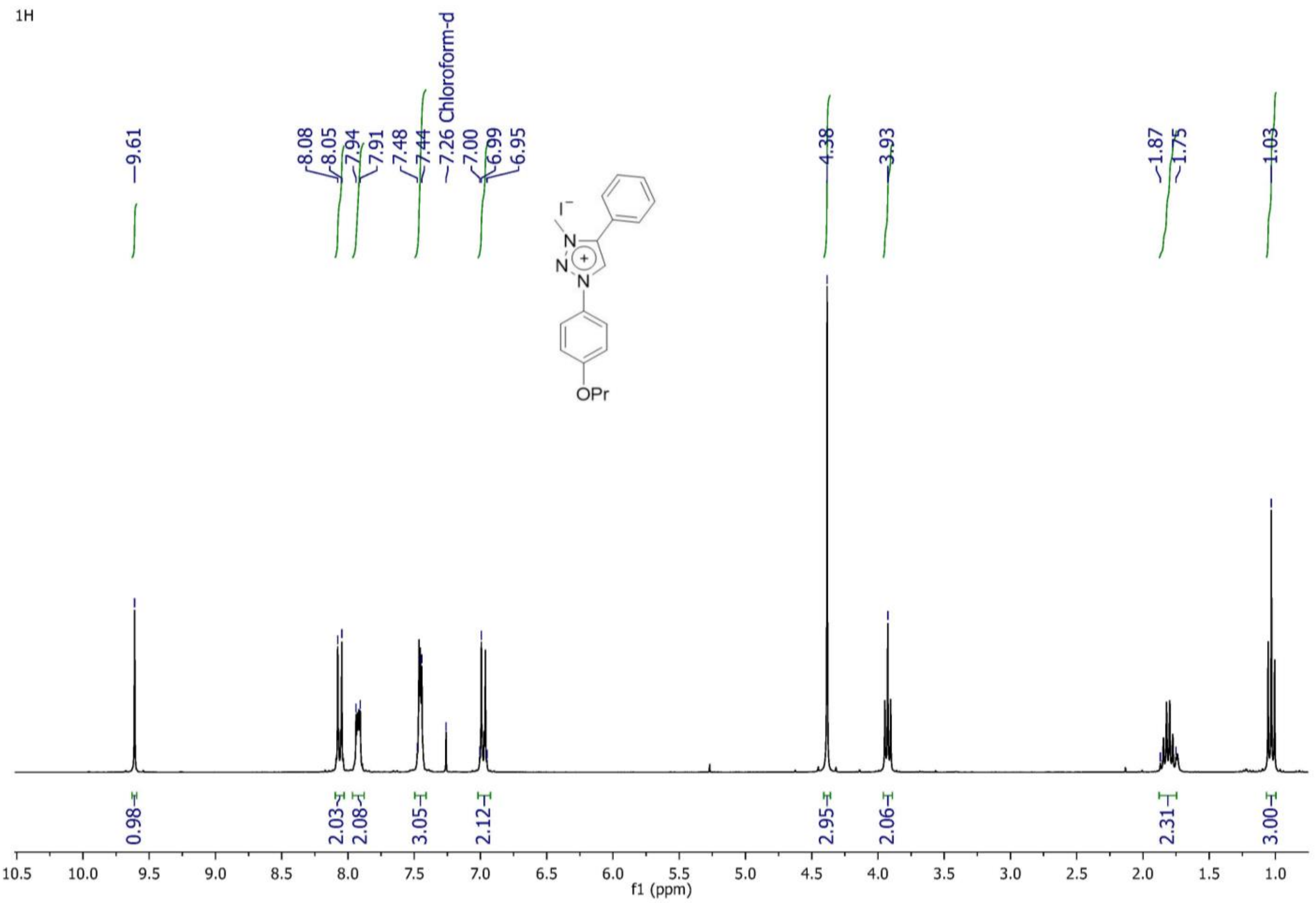

Figure S9: ^{1}H NMR spectrum of 3-methyl-4-phenyl-1-(4-propoxyphenyl)-1*H*-1,2,3-triazolium iodide (**4**)

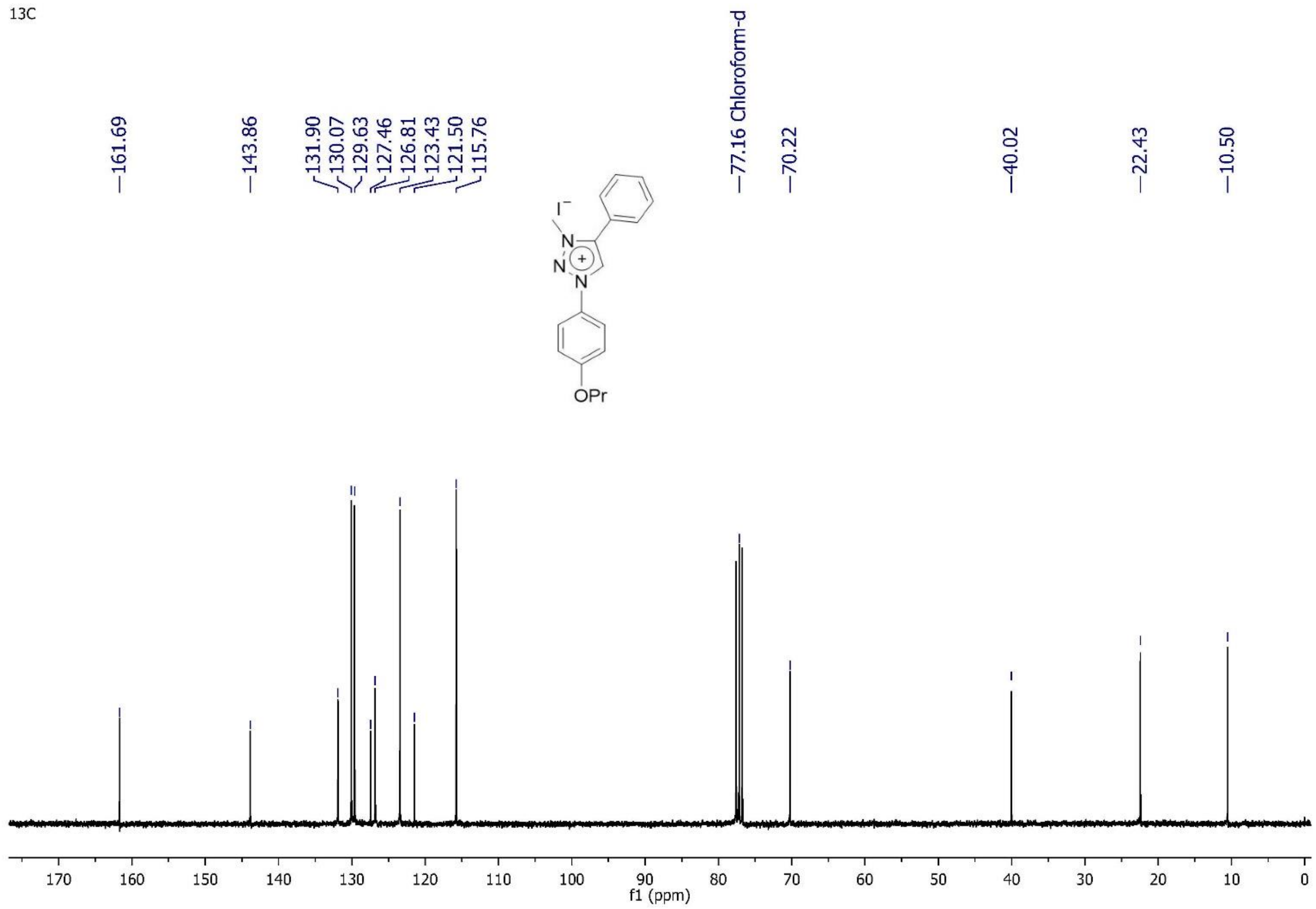

Figure S10: ^{13}C NMR spectrum of 3-methyl-4-phenyl-1-(4-propoxyphenyl)-1*H*-1,2,3-triazolium iodide (**4**)

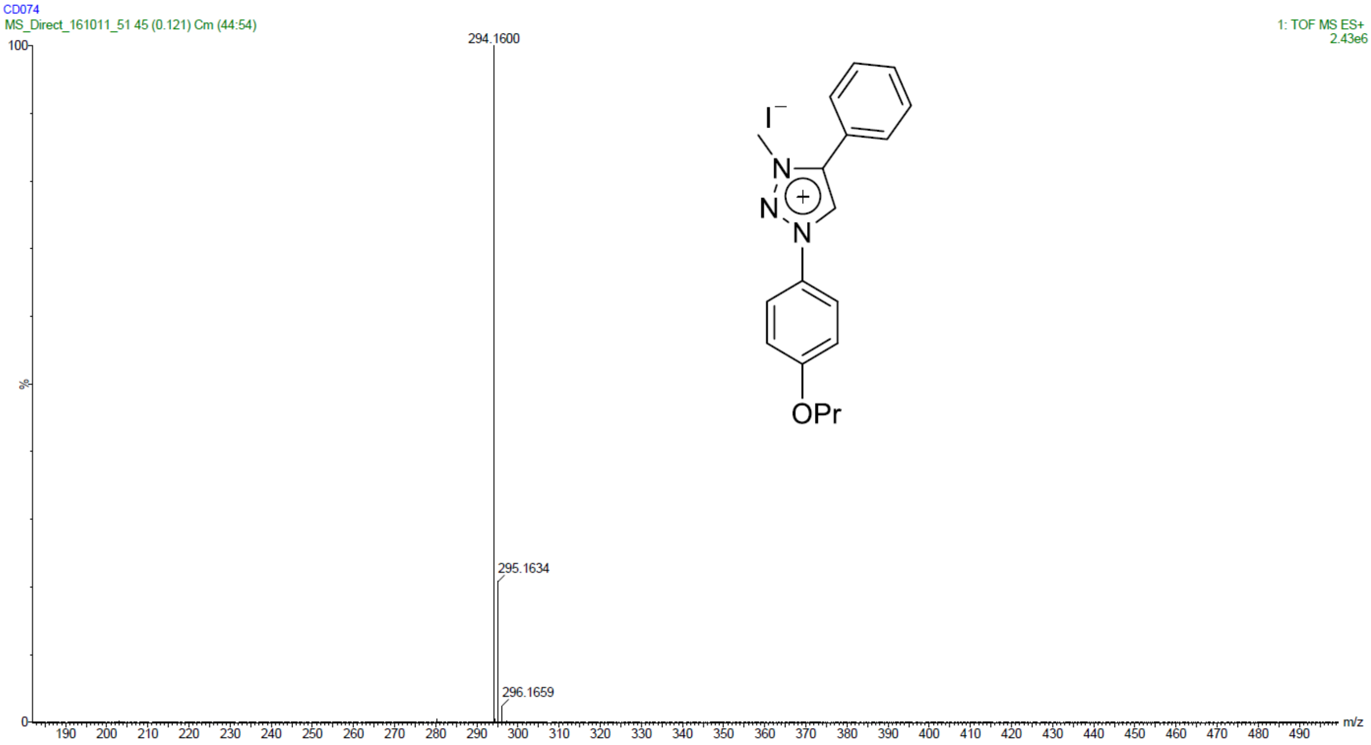

Figure S11: HRMS spectrum for 3-methyl-4-phenyl-1-(4-propoxyphenyl)-1*H*-1,2,3-triazolium iodide (**4**)

(η6-*p*-Cymene)-chloro-(3-methyl-1-(4-propoxyphenyl)-4-phenyl-1,2,3-triazol-5-yl)ruthenium (5)

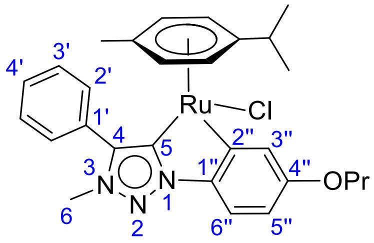

Dry DCM (12 mL) was added to the triazolium salt **4** (90 mg, 0.214 mmol) and Ag_2O (28 mg, 0.117 mmol). The reaction mixture was stirred at rt for 5 hours under a positive pressure of argon and the exclusion of light. The mixture was diluted with DCM and filtered through Celite. Volatiles were removed in vacuo to yield the silver carbene as a white solid (102 mg, 0.193 mmol). The silver carbene was immediately added to a solution of $[RuCl_2(p\text{-cymene})]_2$ (119 mg, 0.193 mmol) in dry DCM (7.0 mL), and the solution was stirred at rt for 21 hours. The reaction mixture was diluted with DCM (10 mL), filtered through Celite and all volatiles removed in vacuo. Further purification was achieved via Al_2O_3 (neutral alumina) flash column chromatography (MeOH/DCM 1:99) affording complex **8** as a brown-orange solid (53 mg, 0.094 mmol, 44%).

R_f (alumina) = 0.45 (MeOH:DCM 1:99)

Mp = 134 – 138 °C

IR (ATR, cm^{-1}): 2960 (C-H), 1559 and 1447 (C=C), 1203 and 1035 (C-O), 1014 (C-N), 800 (C-H)

^{1}H NMR (400 MHz, CHLOROFORM-*d*) δ ppm 7.90 – 7.88 (m, 2H, Ar$H_{2'}$), 7.78 (d, 1H, $^4J_{HH}$ = 2.5 Hz, Ar$H_{3''}$), 7.61 – 7.54 (m, 3H, Ar$H_{3'+4'}$), 7.50 (d, 1H, $^3J_{HH}$ = 8.5 Hz, Ar$H_{6''}$), 6.55 (dd, 1H, $^3J_{HH}$ = 8.5 Hz, $^4J_{HH}$ = 2.5 Hz, Ar$H_{5''}$), 5.25 – 5.22 (m, 2H, ArH_{cym}), 4.81 – 4.77 (m, 2H, ArH_{cym}), 4.07 (s, 3H, NC$H_{3(H6)}$), 4.06 – 3.94 (m, 2H, OCH_2CH$_2$), 2.13 (sept., 1H, $^3J_{HH}$ = 6.9 Hz, C*H*(CH$_3$)$_2$), 1.94 (s, 3H, C$H_{3(cym)}$), 1.89 – 1.80 (m, 2H, CH$_2$CH_2CH$_3$), 1.08 (t, 3H, $^3J_{HH}$ = 7.3 Hz, CH$_2$CH$_2$CH_3), 0.80 (d, 3H, $^3J_{HH}$ = 6.9 Hz, CH(CH_3)$_2$), 0.72 (d, 3H, $^3J_{HH}$ = 6.9 Hz, CH(CH_3)$_2$)

^{13}C{^{1}H} (400 MHz, CHLOROFORM-*d*) δ ppm 171.3 (ArC_5),† 168.2 (Ar$C_{2''}$), 157.7 (Ar$C_{4''}$), 145.7 (ArC_4), 138.9 (Ar$C_{1''}$), 130.8 (Ar$C_{2'}$), 129.8 (Ar$C_{3'}$), 128.9 (Ar$C_{4'}$), 127.3 (Ar$C_{1'}$), 114.4 (Ar*C*), 108.5 (Ar*C*), 102.5 (Ar$C_{cym(quat.)}$), 99.3 (Ar$C_{cym(quat.)}$), 89.6 (Ar*C*H_{cym}), 89.5 (Ar*C*H_{cym}), 88.5 (Ar*C*H_{cym}), 84.6 (Ar*C*H_{cym}), 77.4 (O*C*H$_2$CH$_2$), 69.6 (Ar*C*), 37.0 (N*C*$H_{3(C6)}$), 31.0 (*C*H(CH$_3$)$_2$), 23.1 (CH$_2$*C*H$_2$CH$_3$), 23.0 (CH(*C*H$_3$)$_2$), 21.7 (CH(*C*H$_3$)$_2$), 19.0 (*C*$H_{3(cym)}$), 10.8 (CH$_2$CH$_2$*C*H$_3$)

HRMS–Positive: m/z [M–Cl]$^+$ calcd. for $C_{28}H_{32}N_3ORu$: 528.1597; found: 528.1595.

† The number of ^{13}C NMR resonances matches the expected value, however certain signals have been tentatively assigned. Absolute assignments could not be made without 2D data.

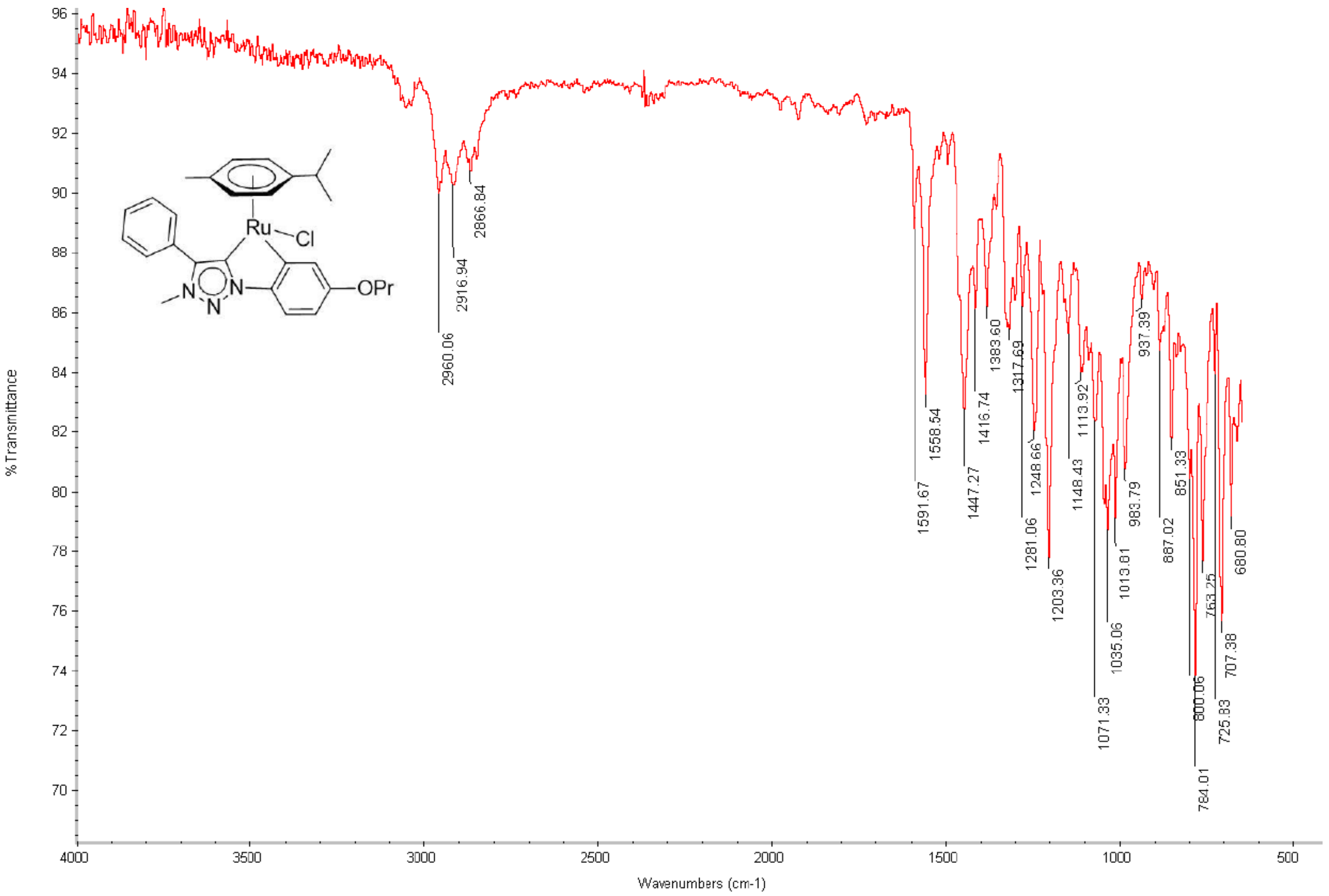

Figure S12: IR spectrum of (η6-*p*-cymene)-chloro-(3-methyl-1-(4-propoxyphenyl)-4-phenyl-1,2,3-triazol-5-yl)ruthenium (**5**)

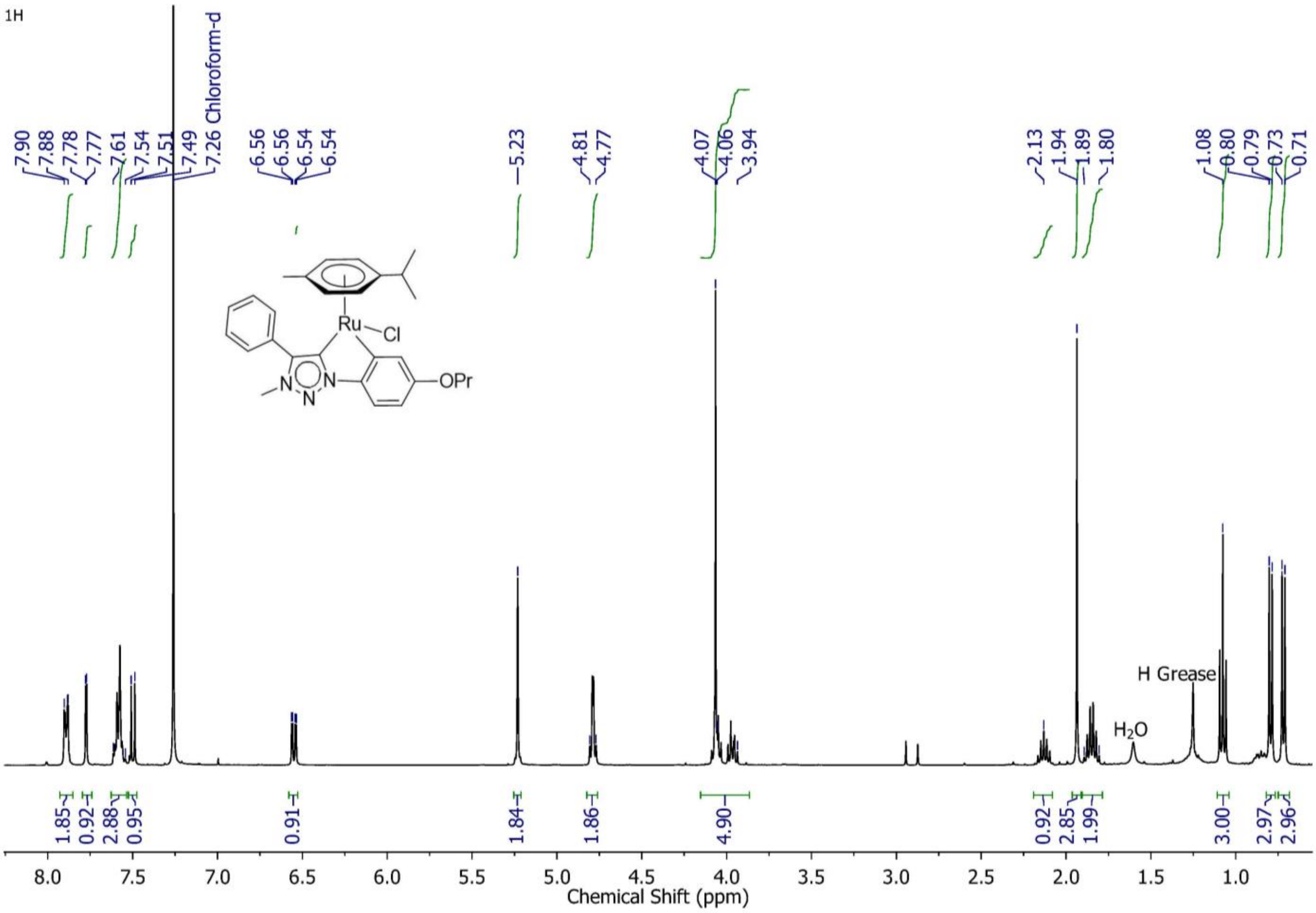

Figure S13: ^{1}H NMR spectrum of (η6-*p*-cymene)-chloro-(3-methyl-1-(4-propoxyphenyl)-4-phenyl-1,2,3-triazol-5-yl)-ruthenium (**5**)

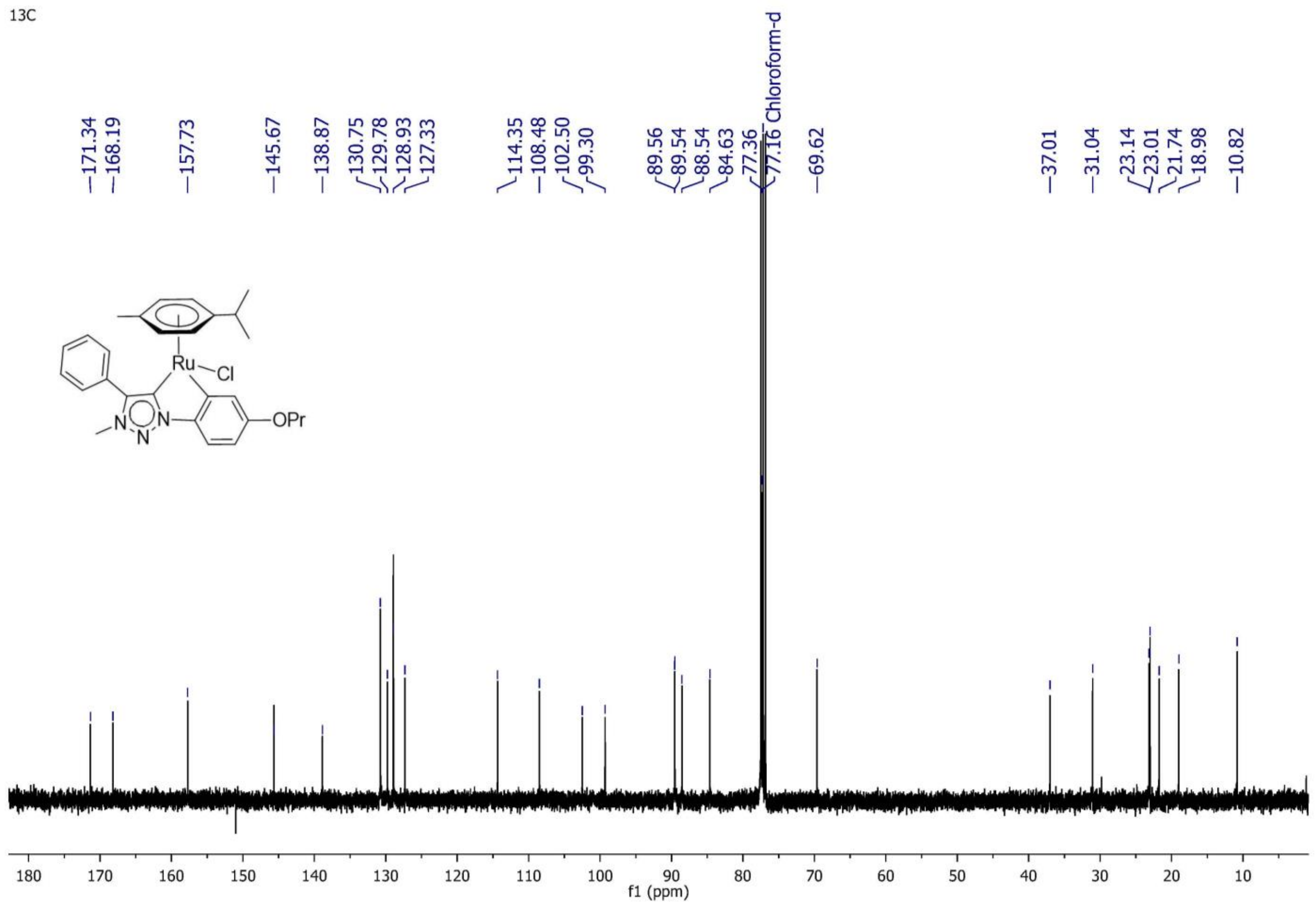

Figure S14: ^{13}C NMR spectrum of (η6-*p*-cymene)-chloro-(3-methyl-1-(4-propoxyphenyl)-4-phenyl-1,2,3-triazol-5-yl)-ruthenium (**5**)

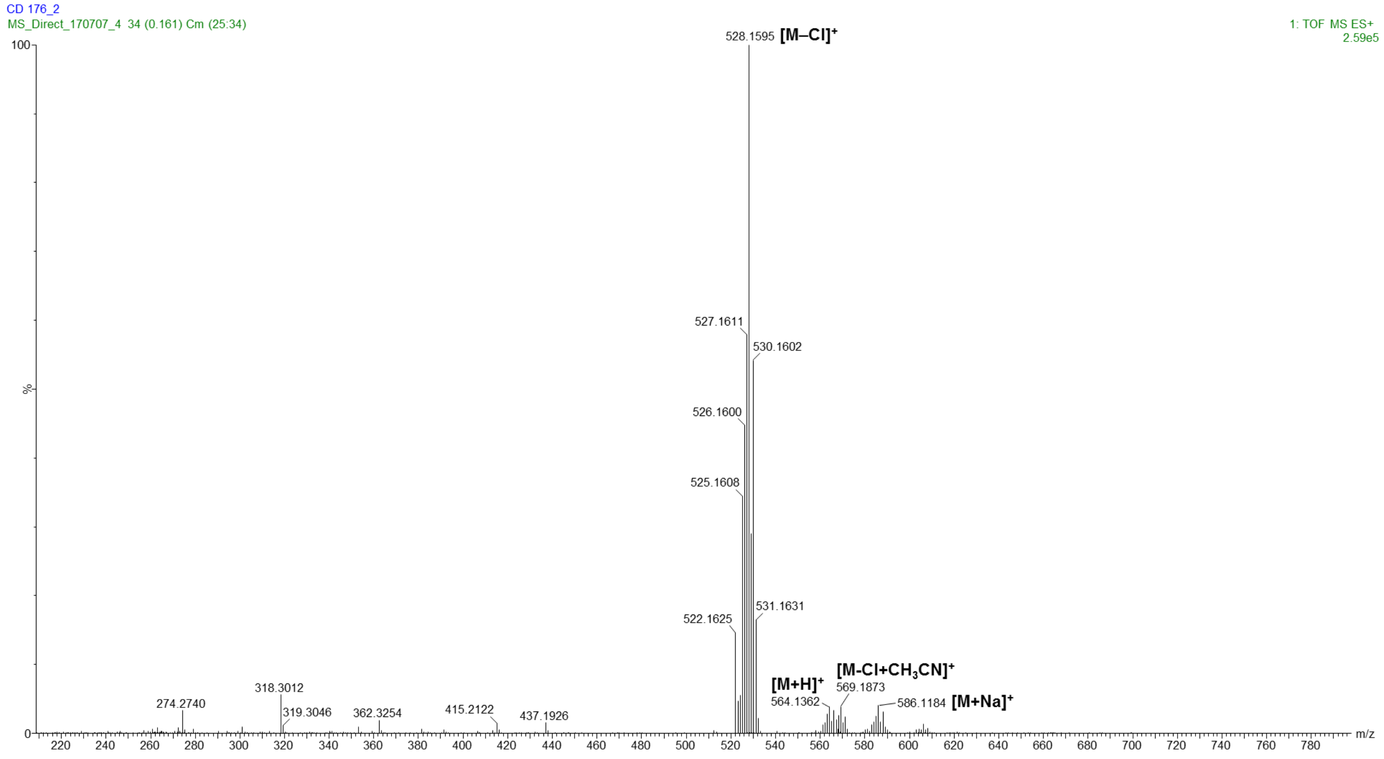

Figure S15: HRMS spectrum of (η6-*p*-cymene)-chloro-(3-methyl-1-(4-propoxyphenyl)-4-phenyl-1,2,3-triazol-5-yl)-ruthenium (**5**)

5-Nitro-25,26,27,28-tetrapropoxycalix[4]arene (9)

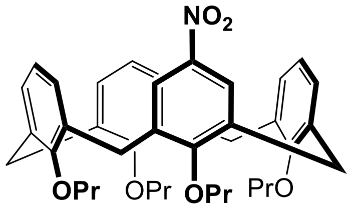

To a solution of tetrapropoxycalix[4]arene (**8**, 1.00 g 1.69 mmol) in a mixture of DCM (100 mL) and sulfuric acid (189 µL, 3.37 mmol) at 0 °C, was added 70% nitric acid (107 µL, 1.69 mmol). The mixture was stirred at 0 °C for 5 mins, followed by stirring at 20 °C for 1 hour and 20 minutes. The reaction was quenched by addition of water (100 mL), and the product was extracted with DCM (3 × 25 mL). The combined organic layers were washed with water (3 × 25 mL) and a saturated sodium bicarbonate solution (3 × 25 mL), after which the organic layer was dried over $MgSO_4$ and the solvent removed in vacuo. Further purification was achieved via silica gel flash column chromatography (DCM/PET 28:72) which afforded mononitro-calix[4]arene **9** as a yellow crystalline solid (0.436 g, 0.68 mmol, 40% yield).

The characterisation data collected for this compound compared well to literature data.[5]

^{1}H NMR (300 MHz, CHLOROFORM-*d*) δ ppm 7.12 (s, 2H, Ar*H*), 6.97 – 6.81 (m, 6H, Ar*H*), 6.23 (m, 3H, Ar*H*), 4.48 (d [app. t], 2H, $^2J_{HH}$ = 13.7 Hz, ArC$H_{2(ax.)}$Ar), 4.44 (d [app. t], 2H, $^2J_{HH}$ = 13.7 Hz, ArC$H_{2(ax.)}$Ar), 4.02 – 3.85 (m, 4H, OCH_2CH$_2$), 3.84 (t, 2H, $^3J_{HH}$ = 6.9 Hz, OCH_2CH$_2$), 3.73 (t, 2H, $^3J_{HH}$ = 6.9 Hz, OCH_2CH$_2$), 3.21 (d, 2H, $^2J_{HH}$ = 13.7 Hz, ArC$H_{2(eq.)}$Ar), 3.17 (d, 2H, $^2J_{HH}$ = 13.7 Hz, ArC$H_{2(eq.)}$Ar), 1.96 – 1.82 (m, 8H, CH$_2$CH_2CH$_3$), 1.10 – 1.03 (m, 6H, CH$_2$CH$_2$CH_3), 0.92 (t, 6H, $^3J_{HH}$ = 7.4 Hz, CH$_2$CH$_2$CH_3).

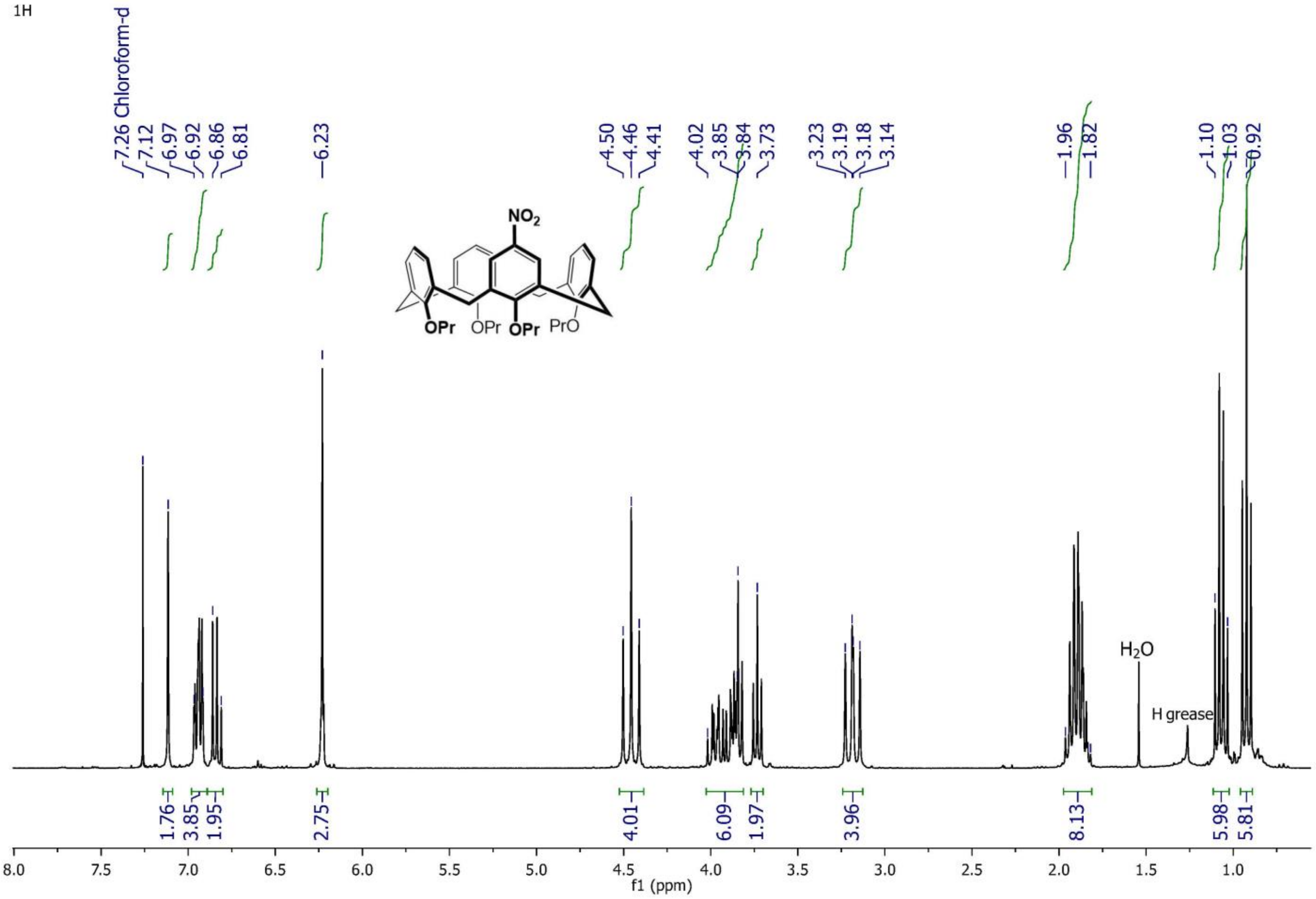

Figure S16: ^{1}H NMR spectrum of 5-nitro-25,26,27,28-tetrapropoxycalix[4]arene (**9**)

5-Amino-25,26,27,28-tetrapropoxycalix[4]arene (10)

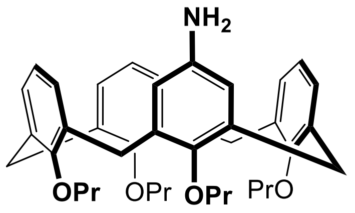

A 2-neck round bottom flask (100 mL) was charged with ethanol (14 mL), mononitro-calix[4]arene **9** (455 mg, 0.713 mmol) and 10% palladium on carbon (114 mg, 0.107 mmol, 0.15 equiv) sequentially. The mixture was heated under reflux and hydrazine hydrate (181 µL, 3.70 mmol, 5.2 equiv) was added. The reaction mixture was heated under reflux for 3 hours after which it was cooled to rt, filtered through Celite, and washed with DCM (20 mL). The solvent was removed in vacuo and the product dried under high vacuum, affording monoaminocalix[4]arene **10** as a white solid (431 mg, 0.71 mmol, 99% yield).

The characterisation data collected for this compound compared well to literature data.[6]

^{1}H NMR (400 MHz, CHLOROFORM-*d*) δ ppm 6.67 – 6.55 (m, 9H, Ar*H*), 5.95 (s, 2H, Ar*H*), 4.47 (d, 2H, $^2J_{HH}$ = 13.4 Hz, ArC*H*$_{2(ax.)}$Ar), 4.38 (d, 2H, $^2J_{HH}$ = 13.4 Hz, ArC*H*$_{2(ax.)}$Ar), 3.87 – 3.73 (m, 8H, OC*H*$_2$CH$_2$), 3.16 (d, 2H, $^2J_{HH}$ = 13.4 Hz, ArC*H*$_{2(eq.)}$Ar), 3.03 (d, 2H, $^2J_{HH}$ = 13.4 Hz, ArC*H*$_{2(eq.)}$Ar), 2.94 (v. br. s., 2H, N*H*$_2$), 1.98 – 1.83 (m, 8H, CH$_2$C*H*$_2$CH$_3$), 1.02 – 0.96 (m, 12H, CH$_2$CH$_2$C*H*$_3$).

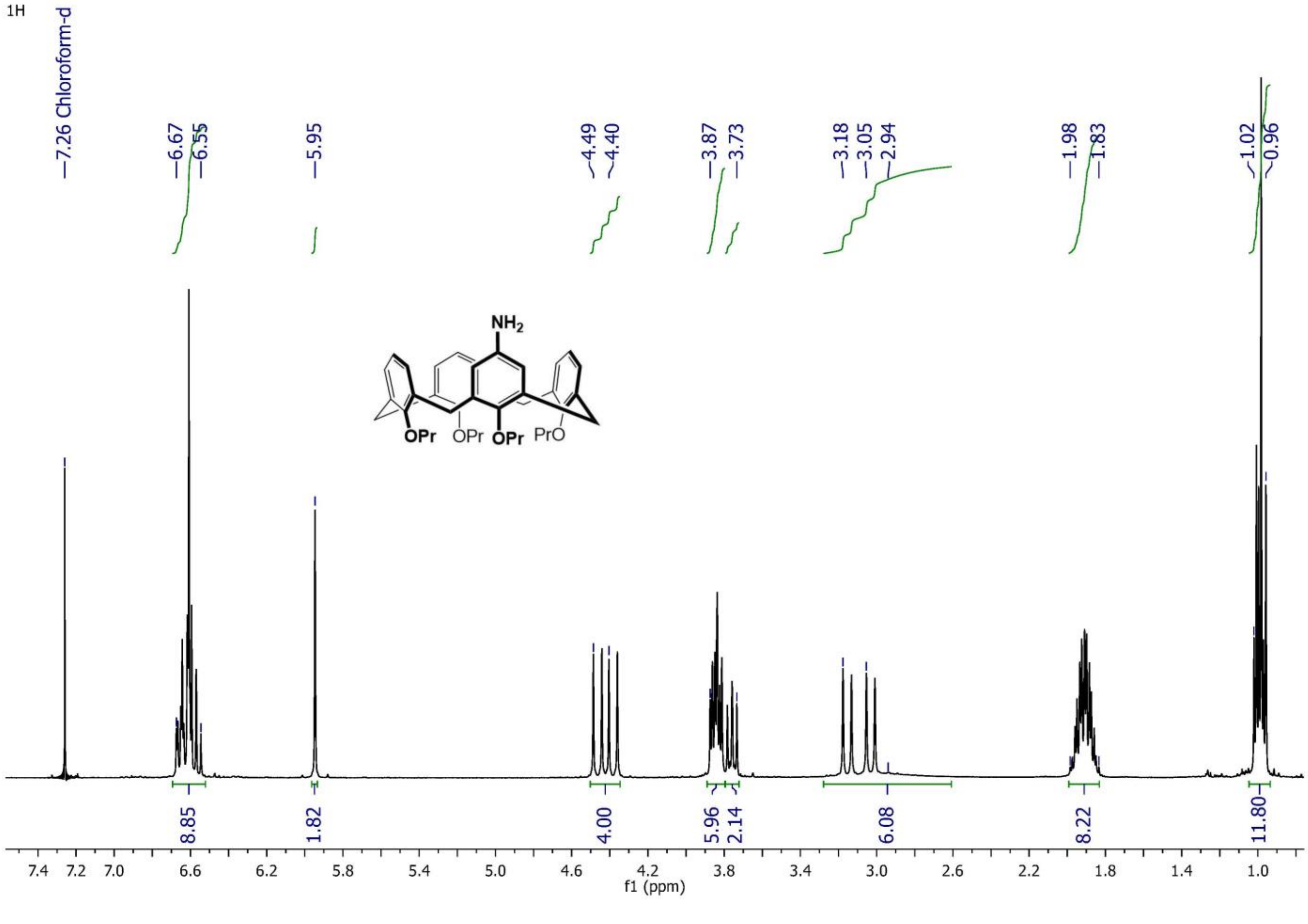

Figure S17: ^{1}H NMR spectrum of 5-amino-25,26,27,28-tetrapropoxycalix[4]arene (**10**)

5-Azido-25,26,27,28-tetrapropoxycalix[4]arene (7)

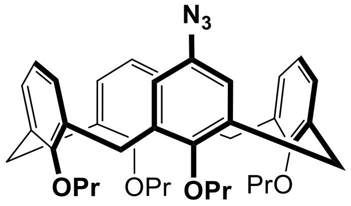

95-97% H_2SO_4 (105 µL, 1.97 mmol, 3.0 equiv) was added to a solution of $NaNO_2$ (182 mg, 2.63 mmol, 4.0 equiv) in H_2O (1.0 mL) at 0 °C. Bubbling of the solution was visible and a brown gas formed immediately upon addition of the acid. The solution of $NaNO_2$ and H_2SO_4 was added dropwise to a solution of monoaminocalix[4]arene **10** (400 mg, 0.658 mmol) in THF (2.0 mL) and MeCN (2.0 mL) at 0 °C, over the space of 5 minutes. The colour of the solution changed to a dark orange after 15 minutes, after which NaN_3 (129 mg, 1.97 mmol, 3.0 equiv) in H_2O (0.5 mL) was added dropwise over 5 minutes. The solution immediately became

a murky peach colour, with bubbles of N_2 evolving. The reaction mixture was stirred for 2 hours while warming to room temperature, after which H_2O (10 mL) was added to quench the reaction. The product was extracted with EtOAc (3 × 20 mL) and the combined organic layers dried over $MgSO_4$. The solvent was removed in vacuo, yielding monoazido-calix[4]arene **13** as an orange solid (409 mg, 0.645 mmol, 98% yield).

R$_f$ **=** 0.64 (DCM:PET 60:40)

Mp = 90 – 94 °C

IR (ATR, cm^{-1}): 3053 (C-H), 2110 (N=N=N), 1455 (C=C), 1264 (C-O), 732 (C-H)

^{1}H NMR (400 MHz, CHLOROFORM-*d*) δ ppm 6.85 (d, 2H, $^3J_{HH}$ = 7.4 Hz, Ar*H*), 6.82 (d, 2H, $^3J_{HH}$ = 7.4 Hz, Ar*H*), 6.74 (t, 2H, $^3J_{HH}$ = 7.4 Hz, Ar*H*), 6.48 (t, 1H, $^3J_{HH}$ = 7.4 Hz, Ar*H*), 6.40 (d, 2H, $^3J_{HH}$ = 7.4 Hz, Ar*H*), 5.97 (s, 2H, Ar*H*), 4.45 (d, 2H, $^2J_{HH}$ = 13.4 Hz, ArC$H_{2(ax.)}$Ar), 4.43 (d, 2H, $^2J_{HH}$ = 13.4 Hz, ArC$H_{2(ax.)}$Ar), 3.94 – 3.86 (m, 4H, OCH_2CH$_2$), 3.77 (t, 2H, $^3J_{HH}$ = 7.0 Hz, OCH_2CH$_2$), 3.75 (t, 2H, $^3J_{HH}$ = 7.0 Hz, OCH_2CH$_2$), 3.16 (d, 2H, $^2J_{HH}$ = 13.4 Hz, ArC$H_{2(eq.)}$Ar), 3.11 (d, 2H, $^2J_{HH}$ = 13.4 Hz, ArC$H_{2(eq.)}$Ar), 1.94 – 1.85 (m, 8H, CH$_2$CH_2CH$_3$), 1.06 – 1.03 (m, 6H, CH$_2$CH$_2$CH_3), 0.94 (t, 6H, $^3J_{HH}$ =7.4 Hz, CH$_2$CH$_2$CH_3)

^{13}C{^{1}H} (400 MHz, CHLOROFORM-*d*) δ ppm 157.4 (Ar*C*), 156.2 (Ar*C*), 153.72 (Ar*C*), 136.4 (Ar*C*), 136.3 (Ar*C*), 135.5 (Ar*C*), 134.5 (Ar*C*), 133.1 (Ar*C*), 129.0 (Ar*C*), 128.5 (Ar*C*), 127.9 (Ar*C*), 122.13 (Ar*C*), 121.9 (Ar*C*), 118.4 (Ar*C*), 77.0 (O*C*H$_2$CH$_2$), 76.9 (O*C*H$_2$CH$_2$), 76.8 (O*C*H$_2$CH$_2$), 31.2 (Ar*C*H$_2$Ar), 31.1 (Ar*C*H$_2$Ar), 23.5 (CH$_2$*C*H$_2$CH$_3$), 23.4 (CH$_2$*C*H$_2$CH$_3$), 23.3 (CH$_2$*C*H$_2$CH$_3$), 10.7 (CH$_2$CH$_2$*C*H$_3$), 10.7 (CH$_2$CH$_2$*C*H$_3$), 10.2 (CH$_2$CH$_2$*C*H$_3$)

HRMS–Positive: m/z $[M+Na]^+$ calcd. for $C_{40}H_{47}N_3O_4Na$: 656.3464; found: 656.3476.

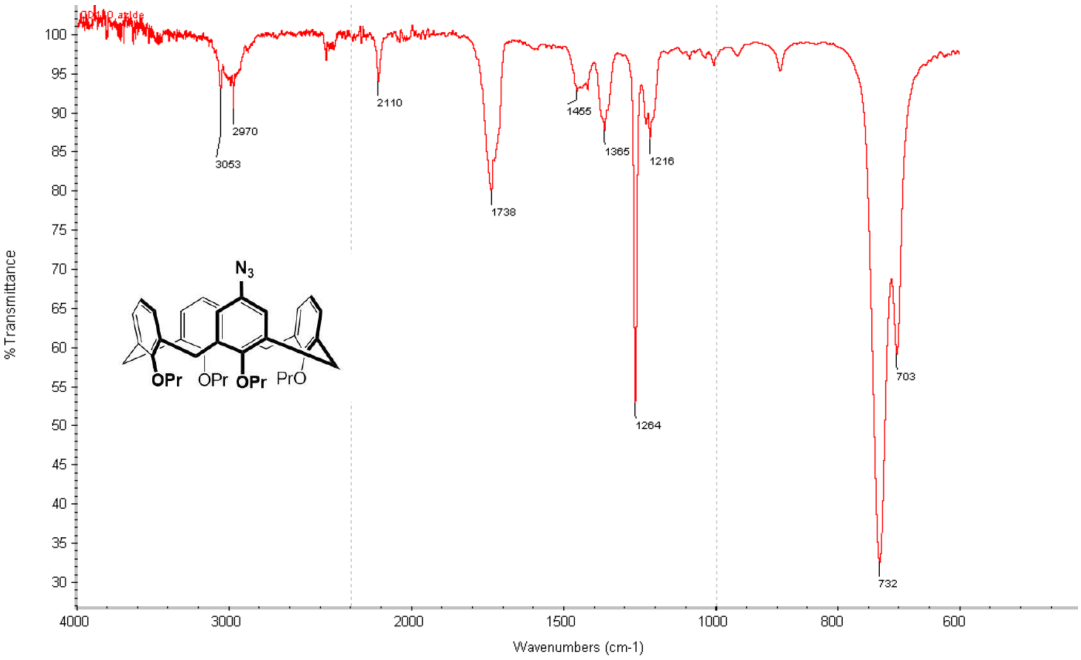

Figure S18: IR spectrum of 5-azido-25,26,27,28-tetrapropoxycalix[4]arene (**7**)

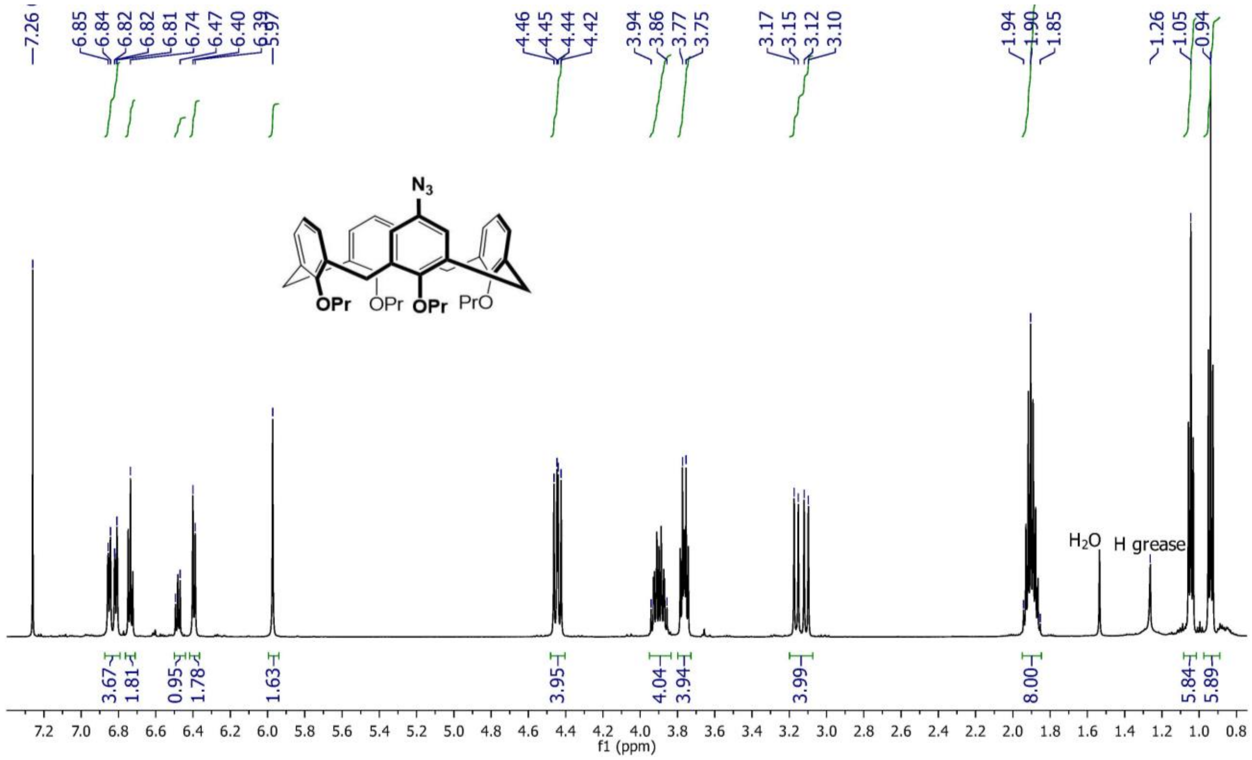

Figure S19: ^{1}HNMR spectrum of 5-azido-25,26,27,28-tetrapropoxycalix[4]arene (**7**)

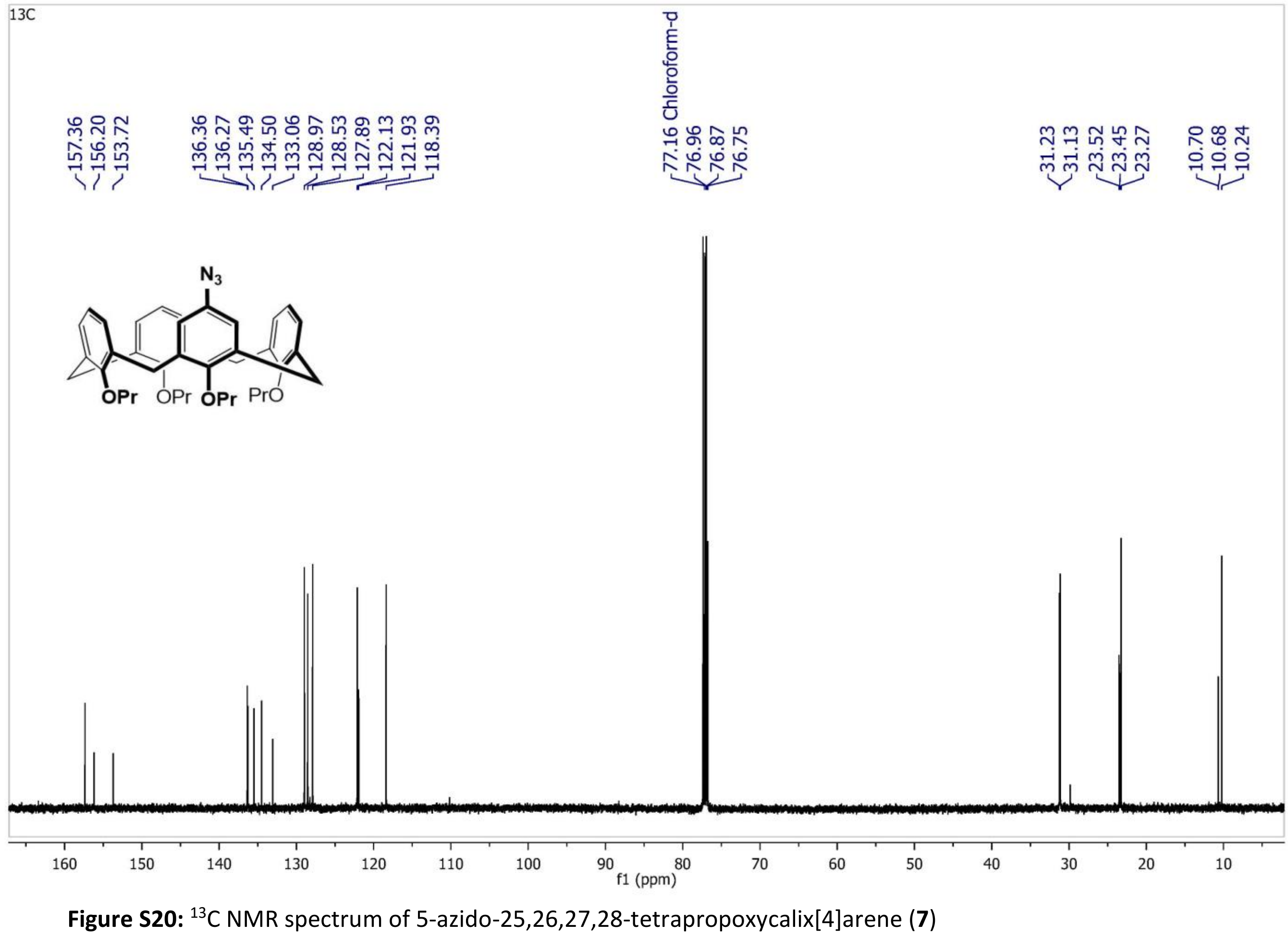

Figure S20: ^{13}C NMR spectrum of 5-azido-25,26,27,28-tetrapropoxycalix[4]arene (**7**)

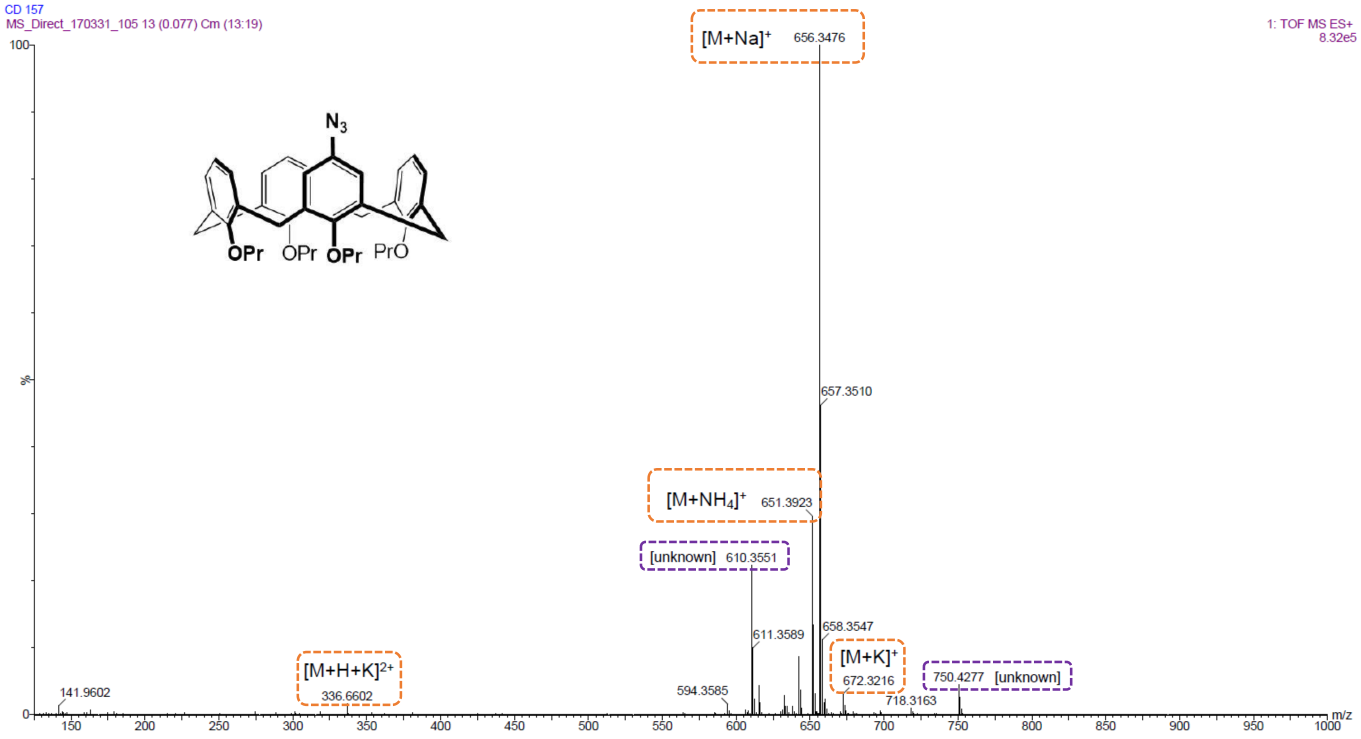

Figure S21: HRMS spectrum of 5-azido-25,26,27,28-tetrapropoxycalix[4]arene (**7**). Various attempts were made to assign the unknown signals, but the predicted formulas made no sense based on possible structures, but probably represent unusual adducts.

5-(4-Phenyl-1*H*-1,2,3-triazole)-25,26,27,28-tetrapropoxycalix[4]arene (11)

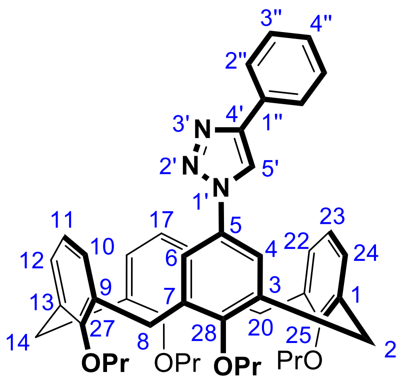

Azidocalix[4]arene **7** (156 mg, 0.246 mmol), sodium ascorbate (21 mg, 0.10 mmol, 0.42 equiv), $CuSO_4{\cdot}5H_2O$ (13 mg, 0.052 mmol, 0.21 equiv) and phenylacetylene (81.0 µL, 0.738 mmol, 3.0 equiv) were added to a vial containing toluene (3.0 mL) and H_2O (0.8 mL). The vial was placed under an inert atmosphere of argon, sealed, and stirred at 60 °C for 24 hours before being cooled to rt. Saturated NH_4Cl (10 mL) was added and the product was extracted with EtOAc (2 × 30 mL). The combined organic layers were dried over $MgSO_4$ and the solvent removed in vacuo, yielding the crude product as a brown solid. Further purification was achieved via silica gel flash column chromatography (gradient, 5:95 to 20:80 EtOAc/PET), yielding calix[4]arene **11** as a yellowish orange solid (158 mg, 0.214 mmol, 87% yield).

***R**$_f$* = 0.67 (EtOAc:PET 60:40)

Mp = 102 – 105 °C

IR (ATR, cm^{-1}): 2959 (C-H), 1585 and 1452 (C=C), 1192 and 1034 (C-O), 1003 (C-N), 756 (C-H)

^{1}H NMR (400 MHz, CHLOROFORM-*d*) δ ppm 7.85 – 7.83 (m, 2H, Ar$H_{2''}$), 7.57 (s, 1H, Ar$H_{5'}$), 7.44 (t, 2H, $^3J_{HH}$ = 7.3 Hz, Ar$H_{3''}$), 7.34 (tt, 1H, $^3J_{HH}$ = 7.3 Hz, $^4J_{HH}$ = 1.1 Hz, Ar$H_{4''}$), 6.96 – 6.94 (m, 4H, Ar$H_{10+24,\ 12+22}$), 6.82 (t, 2H, $^3J_{HH}$ = 7.4 Hz, ArH_{11+23}), 6.63 (s, 2H, ArH_{4+6}), 6.25 (d, 2H, $^3J_{HH}$ = 7.5 Hz, ArH_{16+18}), 6.01 (t, 1H, $^3J_{HH}$ = 7.5 Hz, ArH_{17}), 4.55 (d, 2H, $^2J_{HH}$ = 13.6 Hz, ArC$H_{2(ax.)}$Ar), 4.46 (d, 2H, $^2J_{HH}$ = 13.6 Hz, ArC$H_{2(ax.)}$Ar), 4.03 – 3.90 (m, 4H, OCH_2CH$_2$), 3.81 (t, 2H, $^3J_{HH}$ = 7.0 Hz, OCH_2CH$_2$), 3.74 (t, 2H, $^3J_{HH}$ = 7.0 Hz, OCH_2CH$_2$), 3.24 (d, 2H, $^2J_{HH}$ = 13.6 Hz, ArC$H_{2(eq.)}$Ar), 3.17 (d, 2H, $^2J_{HH}$ = 13.6 Hz, ArC$H_{2(eq.)}$Ar), 2.00 – 1.85 (m, 8H, CH$_2$CH_2CH$_3$), 1.09 (t, 3H, $^3J_{HH}$ = 7.4 Hz, CH$_2$CH$_2$CH_3), 1.06 (t, 3H, $^3J_{HH}$ = 7.4 Hz, CH$_2$CH$_2$CH_3), 0.94 (t, 6H, $^3J_{HH}$ = 7.4 Hz, CH$_2$CH$_2$CH_3)

^{13}C{^{1}H} (400 MHz, CHLOROFORM-*d*) δ ppm 157.5 (Ar*C*), 156.5 (Ar*C*), 156.0 (Ar*C*), 147.7 (Ar*C*), 136.6 (Ar*C*), 136.2 (Ar*C*), 135.6 (Ar*C*), 134.2 (Ar*C*), 131.5 (Ar*C*), 130.7 (Ar*C*), 129.3 (Ar*C*), 129.0 (Ar*C*), 128.7 (Ar*C*), 128.3 (Ar*C*), 127.7 (Ar*C*), 125.9

(ArC), 122.4 (ArC), 121.9 (ArC), 120.2 (ArC), 117.8 (ArC), 77.2 (OCH_2CH_2), 77.0 (OCH_2CH_2), 76.8 (OCH_2CH_2), 31.3 (ArCH_2Ar), 31.1 (ArCH_2Ar), 23.6 ($CH_2CH_2CH_3$), 23.5 ($CH_2CH_2CH_3$), 23.2 ($CH_2CH_2CH_3$), 10.8 ($CH_2CH_2CH_3$), 10.8 ($CH_2CH_2CH_3$), 10.2 ($CH_2CH_2CH_3$)

HRMS–Positive: m/z $[M+Na]^+$ calcd. for $C_{48}H_{53}N_3O_4Na$: 758.3933; found: 758.3926.

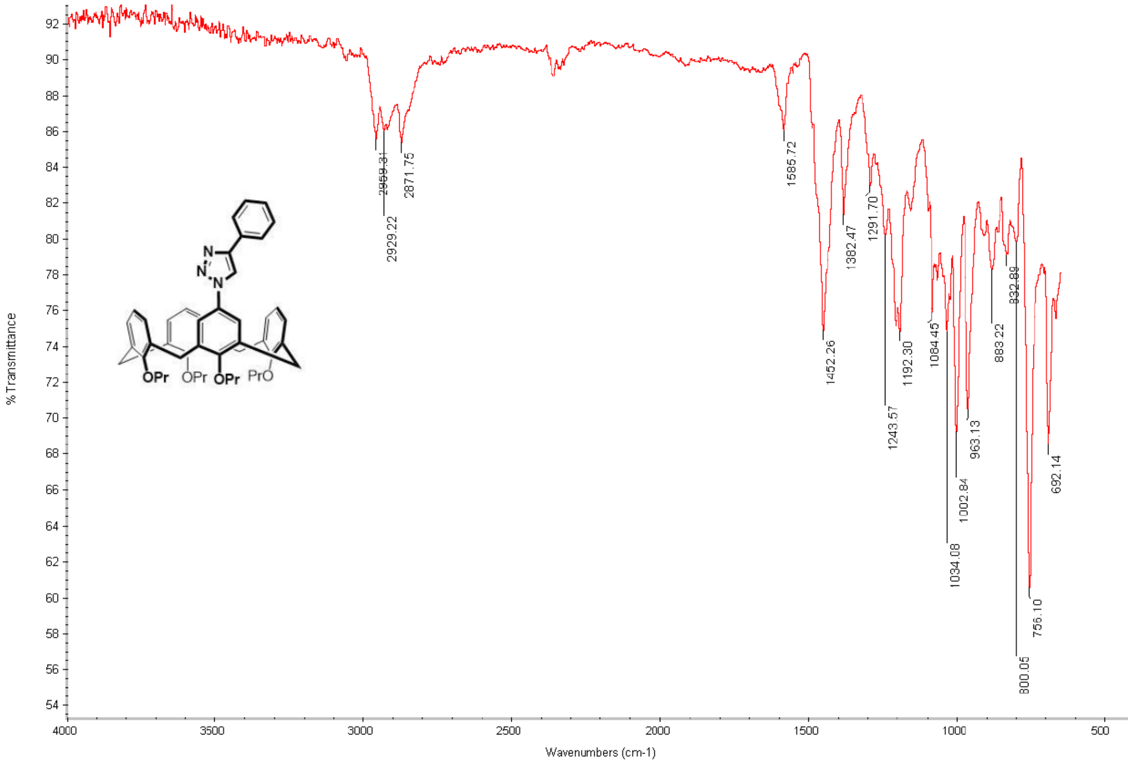

Figure S22: IR spectrum of 5-(4-phenyl-1*H*-1,2,3-triazole)-25,26,27,28-tetrapropoxycalix[4]arene (**11**)

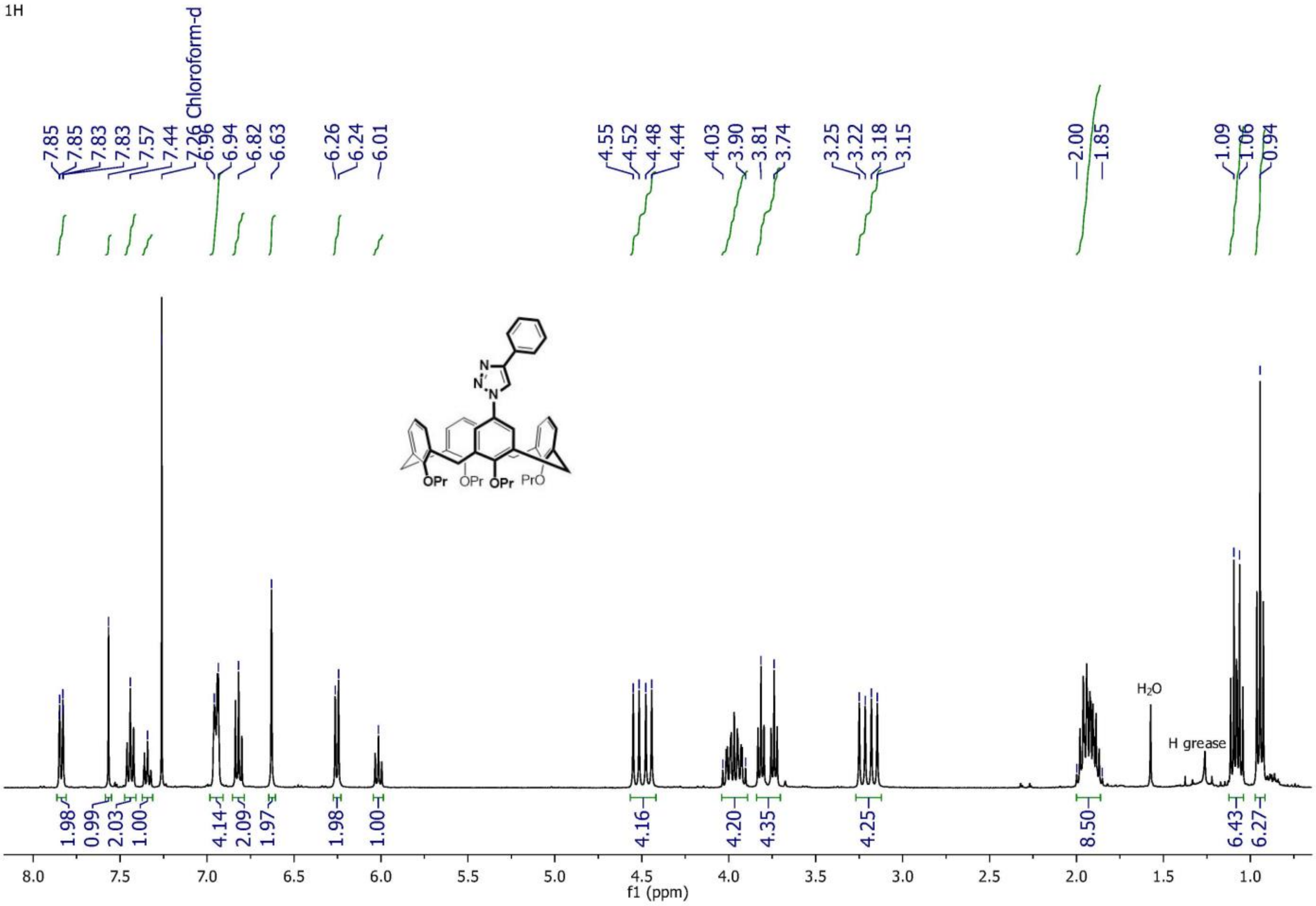

Figure S23: ^{1}H NMR spectrum of 5-(4-phenyl-1*H*-1,2,3-triazole)-25,26,27,28-tetrapropoxycalix[4]arene (**11**)

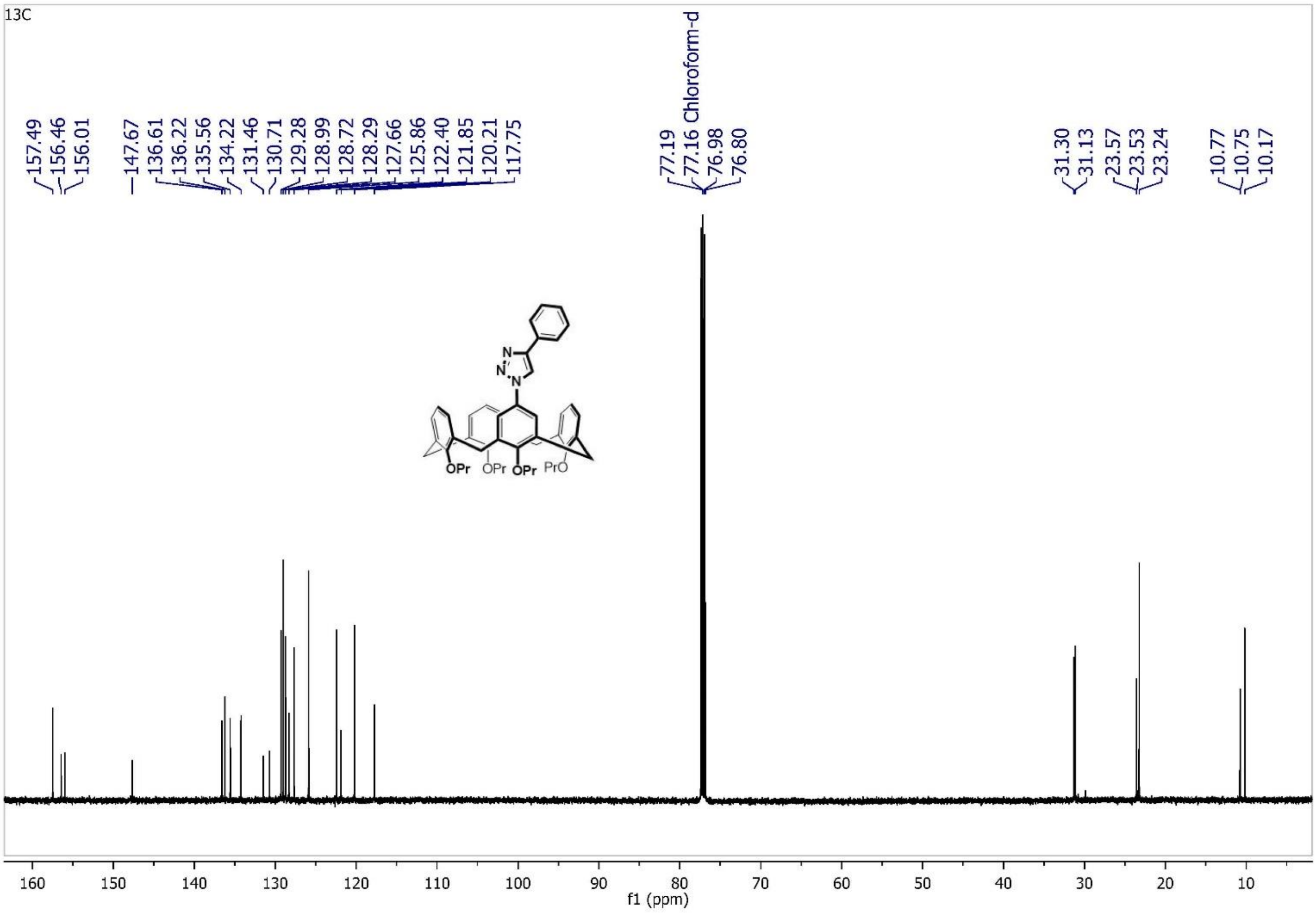

Figure S24: ^{13}C NMR spectrum of 5-(4-phenyl-1*H*-1,2,3-triazole)-25,26,27,28-tetrapropoxycalix[4]arene (**11**)

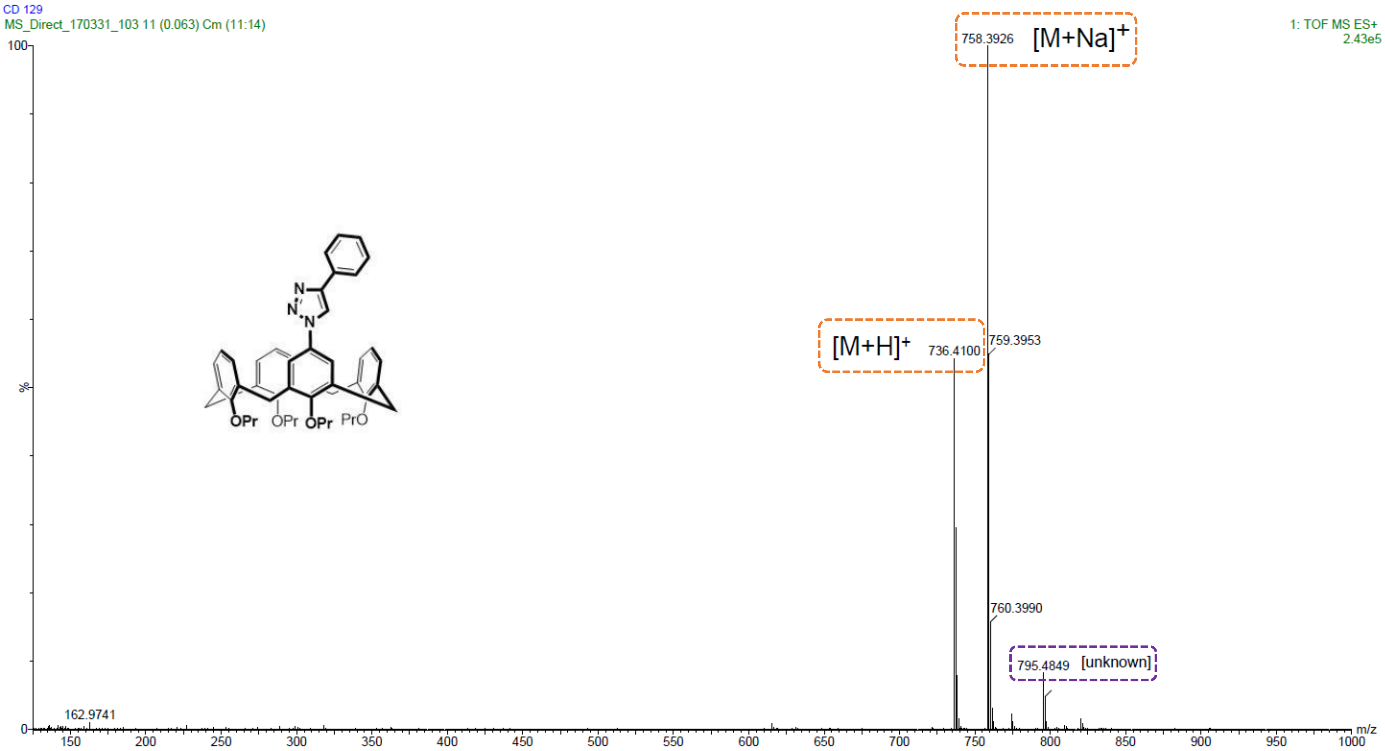

Figure S25: HRMS spectrum of 5-(4-phenyl-1*H*-1,2,3-triazole)-25,26,27,28-tetrapropoxycalix[4]arene (**11**). Unknown fits with $[M+C_3H_{10}N]^+$ which could be a number of ammonium isomers (e.g., trimethylammonium), but it is not clear where this came from.

5-(3-Methyl-4-phenyl-1*H*-1,2,3-triazol-3-ium)-25,26,27,28-tetrapropox-calix[4]arene iodide (12)

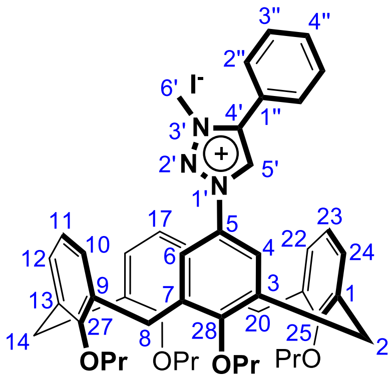

Calix[4]arene **11** (200 mg, 0.271 mmol) was dissolved in MeCN (4.0 mL) in a sealable vial, MeI (423 µL, 6.79 mmol, 25 equiv) was added, and the vial sealed. The clear orange mixture was stirred under an inert atmosphere of argon for 4 days at 80 °C, resulting in a dark brown solution, which was cooled to rt. The solvent was removed in vacuo and purification of the product was achieved via silica gel flash column chromatography (gradient, DCM to MeOH/DCM 10:90). Calix[4]arene **12** was obtained as an orange crystalline solid (237 mg, 0.270 mmol, 99% yield).

$\boldsymbol{R_f}$ = 0.67 (MeOH:DCM 9:91);

Mp = 141 – 145 °C

IR (ATR, cm^{-1}): 2957 (C-H), 1584 and 1454 (C=C), 1194 and 1036 (C-O), 1003 (C-N), 759 (C-H)

^{1}H NMR (400 MHz, CHLOROFORM-*d*) δ ppm 8.92 (s, 1H, Ar$H_{5'}$), 7.90 – 7.89 (m, 2H, Ar$H_{2''}$), 7.43 (t, 2H, $^{3}J_{HH}$ = 7.3 Hz, Ar$H_{3''}$), 7.38 – 7.35 (m, 1H, Ar$H_{4''}$), 7.12 (s, 2H, ArH_{4+6}), 6.95 (dd, 2H, $^{3}J_{HH}$ = 7.4 Hz, $^{4}J_{HH}$ = 1.3 Hz, ArH_{10+24}), 6.82 (dd, 2H, $^{3}J_{HH}$ = 7.4 Hz, $^{4}J_{HH}$ = 1.3 Hz, ArH_{12+22}), 6.74 (t, 2H, $^{3}J_{HH}$ = 7.4 Hz, ArH_{11+23}), 6.40 (d, 2H, $^{3}J_{HH}$ = 7.5 Hz, ArH_{16+18}), 6.13 (t, 1H, $^{3}J_{HH}$ = 7.5 Hz, ArH_{17}), 4.51 (d, 2H, $^{2}J_{HH}$ = 13.6 Hz, ArC$H_{2+8(ax.)}$Ar), 4.45 (d, 2H, $^{2}J_{HH}$ = 13.6 Hz, ArC$H_{14+20(ax.)}$Ar), 4.38 (s, 3H, $H_{6'}$), 3.97 – 3.92 (m, 2H, OCH_2CH$_2$), 3.90 – 3.86 (m, 4H, OCH_2CH$_2$) 3.77 (t, 2H, $^{3}J_{HH}$ = 7.2 Hz, OCH_2CH$_2$), 3.34 (d, 2H, $^{2}J_{HH}$ = 13.6 Hz, ArC$H_{2+8(eq.)}$Ar), 3.16 (d, 2H, $^{2}J_{HH}$ = 13.6 Hz, ArC$H_{14+20(eq.)}$Ar), 1.97 – 1.87 (m, 8H, CH$_2$CH_2CH$_3$), 1.05 (t, 3H, $^{3}J_{HH}$ = 7.4 Hz, CH$_2$CH$_2$CH_3), 1.02 (t, 3H, $^{3}J_{HH}$ = 7.4 Hz, CH$_2$CH$_2$CH_3), 0.96 (t, 6H, $^{3}J_{HH}$ = 7.4 Hz, CH$_2$CH$_2$CH_3)

^{13}C{^{1}H} (400 MHz, CHLOROFORM-*d*) δ ppm 159.4 (ArC_{28}), 156.9 (ArC_{25+27}), 156.4 (ArC_{26}), 143.7 (Ar$C_{4'}$), 137.8 (ArC_{3+7}), 135.9 (ArC_{13+21}), 134.8 (ArC_{15+19}), 134.4 (ArC_{1+9}), 132.1 (Ar$C_{4''}$), 130.0 (Ar$C_{2''}$), 129.7 (Ar$C_{3''}$), 129.1 (ArC_{12+22}), 128.8 (ArC_{10+24}), 128.7 (ArC_5) 127.8 (ArC_{16+18}), 126.2 (Ar$C_{5'}$), 122.7 (ArC_{11+23}), 121.4 (Ar$C_{1''}$),§ 121.3 (ArC_{17}),‡ 120.9 (ArC_{4+6}), 77.4 (OCH$_2$CH$_2$), 77.0 (OCH$_2$CH$_2$), 76.8 (OCH$_2$CH$_2$), 40.1 ($C_{6'}$), 31.1 (ArC_{2+8}Ar), 31.0 (ArC_{14+20}Ar), 23.4 (CH$_2C$H$_2$CH$_3$), 23.2 (CH$_2C$H$_2$CH$_3$), 10.54 (CH$_2$CH$_2C$H$_3$), 10.48 (CH$_2$CH$_2C$H$_3$), 10.2 (CH$_2$CH$_2C$H$_3$);

^{1}H,^{1}H GCOSY (600/600 MHz, CHLOROFORM-*d*): δ ^{1}H/δ ^{1}H ppm 7.90§ / 7.43 (Ar$H_{2''}$ / Ar$H_{3''}$), 7.43 / 7.90, 7.37 (Ar$H_{3''}$ / Ar$H_{2''}$, Ar$H_{4''}$), 7.37 / 7.90 (Ar$H_{4''}$ / Ar$H_{3''}$), 6.95 / 6.74 (ArH_{10+24} / ArH_{11+23}), 6.82 / 6.74 (ArH_{12+22} / ArH_{11+23}), 6.74 / 6.95, 6.82 (ArH_{11+23} / ArH_{10+24}, ArH_{12+22}), 6.40 / 6.13 (ArH_{16+18} / ArH_{17}), 6.13 / 6.40 (ArH_{17} / ArH_{16+18}), 4.51 / 3.34 (ArC$H_{2+8(ax.)}$Ar / ArC$H_{2+8(eq.)}$Ar), 4.45 / 3.16 (ArC$H_{14+20(ax.)}$Ar / ArC$H_{14+20(eq.)}$Ar), 3.95 / 1.92 (OCH_2CH$_2$ / CH$_2$CH_2CH$_3$), 3.88 / 1.92 (OCH_2CH$_2$ / CH$_2$CH_2CH$_3$), 3.77 / 1.92 (OCH_2CH$_2$ / CH$_2$CH_2CH$_3$), 3.34 / 4.51 (ArC$H_{2+8(eq.)}$Ar / ArC$H_{2+8(ax.)}$Ar), 3.16 / 4.45 (ArC$H_{14+20(eq.)}$Ar / ArC$H_{14+20(ax.)}$Ar), 1.92 / 3.95, 3.88, 3.77, 1.05, 1.02, 0.96 (CH$_2$CH_2CH$_3$ / OCH_2CH$_2$, OCH_2CH$_2$, OCH_2CH$_2$, CH$_2$CH$_2$CH_3, CH$_2$CH$_2$CH_3, CH$_2$CH$_2$CH_3), 1.05 / 1.92 (CH$_2$CH$_2$CH_3 / CH$_2$CH_2CH$_3$), 1.02 / 1.92 (CH$_2$CH$_2$CH_3 / CH$_2$CH_2CH$_3$), 0.96 / 1.92 (CH$_2$CH$_2$CH_3 / CH$_2$CH_2CH$_3$)

^{1}H,^{13}C GHSQC (600/600 MHz, CHLOROFORM-*d*): δ ^{1}H/δ ^{13}C ppm 8.92 / 126.2 (Ar$H_{5'}$ / Ar$C_{5'}$), 7.90 / 130.0 (Ar$H_{2''}$ / Ar$C_{2''}$), 7.43 / 129.7 (Ar$H_{3''}$ / Ar$C_{3''}$), 7.37 / 132.1 (Ar$H_{4''}$ / Ar$C_{4''}$), 7.12 / 120.9 (ArH_{4+6} / ArC_{4+6}), 6.95 / 128.8 (ArH_{10+24} / ArC_{10+24}), 6.82 / 129.1 (ArH_{12+22} / ArC_{12+22}), 6.74 / 122.7 (ArH_{11+23} / ArC_{11+23}), 6.40 / 127.8 (ArH_{16+18} / ArC_{16+18}), 6.13 / 121.3 (ArH_{17} / ArC_{17}), 4.51 / 31.1 (ArC$H_{2+8(ax.)}$Ar / Ar$C_{2+8(ax.)}$Ar), 4.45 / 31.0 (ArC$H_{14+20(ax.)}$Ar / Ar$C_{14+20(ax.)}$Ar), 4.38 / 40.1 (NC$H_{3(H6')}$ / $C_{6'}$), 3.95 / 76.8 (OCH_2CH$_2$ / OCH$_2$CH$_2$), 3.88 / 77.4, 76.8 (OCH_2CH$_2$ / OCH$_2$CH$_2$, OCH$_2$CH$_2$), 3.77 / 77.0 (OCH_2CH$_2$ / OCH$_2$CH$_2$), 3.34 / 31.1 (ArC$H_{2+8(eq.)}$Ar / Ar$C_{2+8(eq.)}$Ar), 3.16 / 31.0 (ArC$H_{14+20(eq.)}$Ar / Ar$C_{14+20(eq.)}$Ar), 1.92 / 23.4, 23.2 (CH$_2$CH_2CH$_3$ / CH$_2C$H$_2$CH$_3$, CH$_2C$H$_2$CH$_3$), 1.05 / 10.48 (CH$_2$CH$_2$CH_3 / CH$_2$CH$_2C$H$_3$), 1.02 / 10.54 (CH$_2$CH$_2$CH_3 / CH$_2$CH$_2C$H$_3$), 0.96 / 10.2 (CH$_2$CH$_2$CH_3 / CH$_2$CH$_2C$H$_3$)

^{1}H,^{13}C GHMBC (600/600 MHz, CHLOROFORM-*d*): δ ^{1}H/δ ^{13}C ppm 8.92 / 143.7 (Ar$H_{5'}$ / Ar$C_{4'}$), 7.90 / 143.7, 132.1, 130.0 (Ar$H_{2''}$ / Ar$C_{4'}$, Ar$C_{4''}$, Ar$C_{2''}$), 7.43 / 130.0, 121.4 (Ar$H_{3''}$ / Ar$C_{2''}$, Ar$C_{1''}$), 7.37 / 130.0 (Ar$H_{4''}$ / Ar$C_{2''}$), 7.12 / 159.4, 137.8,

‡ Signals have been tentatively assigned. Absolute assignments could not be made with the available 2D data.

§ For tidiness, multiplet signals have been reported as single δ ppm values, taken from the center of the multiplet. This was applied to all 2D NMR data.

128.7, 31.1 (ArH_{4+6} / ArC_{28}, ArC_{3+7}, ArC_5, ArC_{2+8}Ar), 6.95 / 156.9, 129.1, 31.1 (ArH_{10+24} / ArC_{25+27}, ArC_{12+22}, ArC_{2+8}Ar), 6.82 / 156.9, 128.8, 31.0 (ArH_{12+22} / ArC_{25+27}, ArC_{10+24}, ArC_{14+20}Ar), 6.74 / 135.9, 134.4 (ArH_{11+23} / ArC_{13+21}, ArC_{1+9}), 6.40 / 156.4, 127.8, 31.0 (ArH_{16+18} / ArC_{26}, ArC_{16+18}, ArC_{14+20}Ar), 6.13 / 134.8 (ArH_{17} / ArC_{15+19}), 4.51 / 159.4, 156.9, 137.8, 134.4, 128.8, 120.9 (ArC$H_{2+8(ax.)}$Ar / ArC_{28}, ArC_{25+27}, ArC_{3+7}, ArC_{1+9}, ArC_{10+24}, ArC_{4+6}), 4.45 / 156.9, 156.4, 135.9, 134.8, 129.1, 127.8 (ArC$H_{14+20(ax.)}$Ar / ArC_{25+27}, ArC_{26}, ArC_{13+21}, ArC_{15+19}, ArC_{12+22}, ArC_{16+18}), 4.38 / 143.7 (NC$H_{3(H6')}$ / $C_{4'}$), 3.95 / 156.9, 23.2, 10.2 (OCH_2CH$_2$ / ArC_{25+27}, CH$_2$$CH_2CH_3$, CH$_2CH_2$$CH_3$), 3.88 / 159.4, 156.9, 23.4, 10.48, 10.2 (OCH_2CH$_2$ / ArC_{28}, ArC_{25+27}, CH$_2$$CH_2CH_3$, CH$_2CH_2$$CH_3$, CH$_2CH_2$$CH_3$), 3.77 / 156.4, 23.4, 10.54 (OCH_2CH$_2$ / ArC_{26}, CH$_2$$CH_2CH_3$, CH$_2CH_2$$CH_3$), 3.34 / 159.4, 156.9, 137.8, 134.4, 128.8, 120.9 (ArC$H_{2+8(eq.)}$Ar / ArC_{28}, ArC_{25+27}, ArC_{3+7}, ArC_{1+9}, ArC_{10+24}, ArC_{4+6}), 3.16 / 156.9, 156.4, 135.9, 134.8, 129.1, 127.8 (ArC$H_{14+20(eq.)}$Ar / ArC_{25+27}, ArC_{26}, ArC_{13+21}, ArC_{15+19}, ArC_{12+22}, ArC_{16+18}), 1.92 / 77.4, 77.0, 76.8, 10.54, 10.48, 10.2 (CH$_2$CH_2CH$_3$ / OCH$_2$CH$_2$, CH$_2$CH$_2$$CH_3$), 1.05 / 77.4, 23.4 (CH$_2CH_2CH_3$ / OCH$_2$CH$_2$, CH$_2$$CH_2CH_3$), 1.02 / 77.0, 23.4 (CH$_2CH_2CH_3$ / OCH$_2$CH$_2$, CH$_2$$CH_2CH_3$), 0.96 / 76.8, 23.2 (CH$_2CH_2CH_3$ / OCH$_2$CH$_2$, CH$_2$$CH_2CH_3$);

HRMS–Positive: m/z [M-I]$^+$ calcd. for $C_{49}H_{56}N_3O_4$: 750.4271; found: 750.4265.

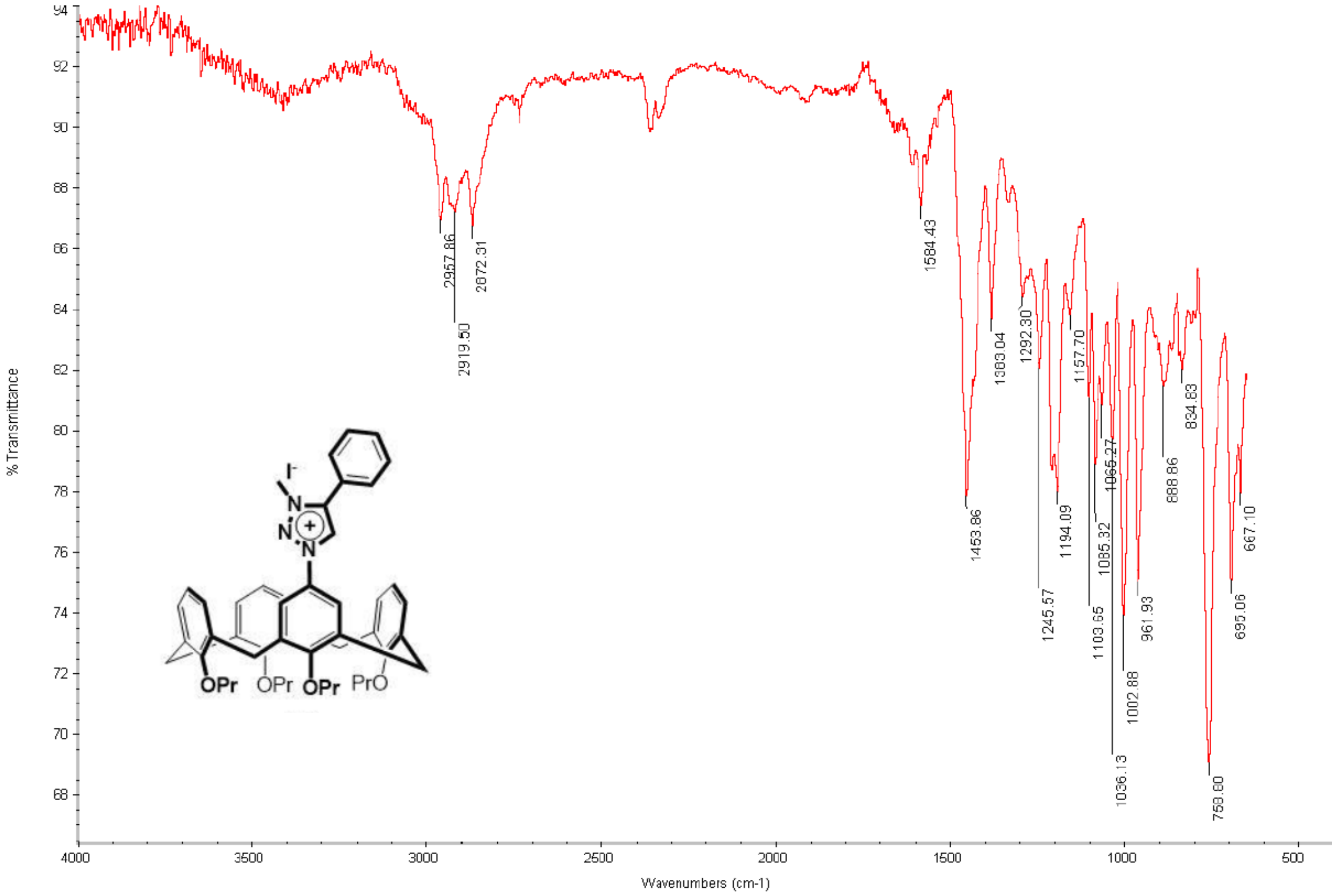

Figure S26: IR spectrum of triazoliumcalix[4]arene (**12**)

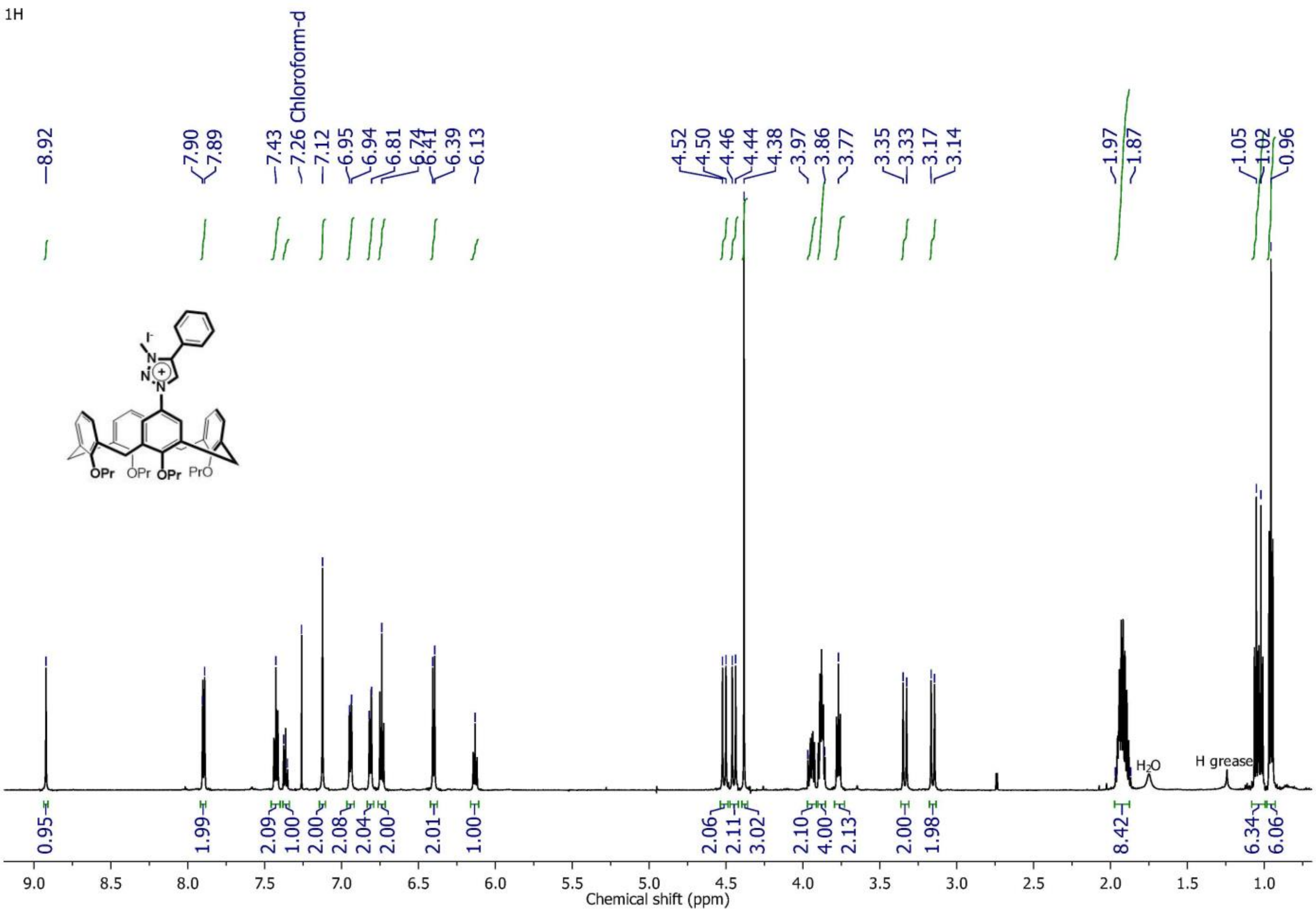

Figure S27: ^{1}H NMR spectrum of triazoliumcalix[4]arene (**12**)

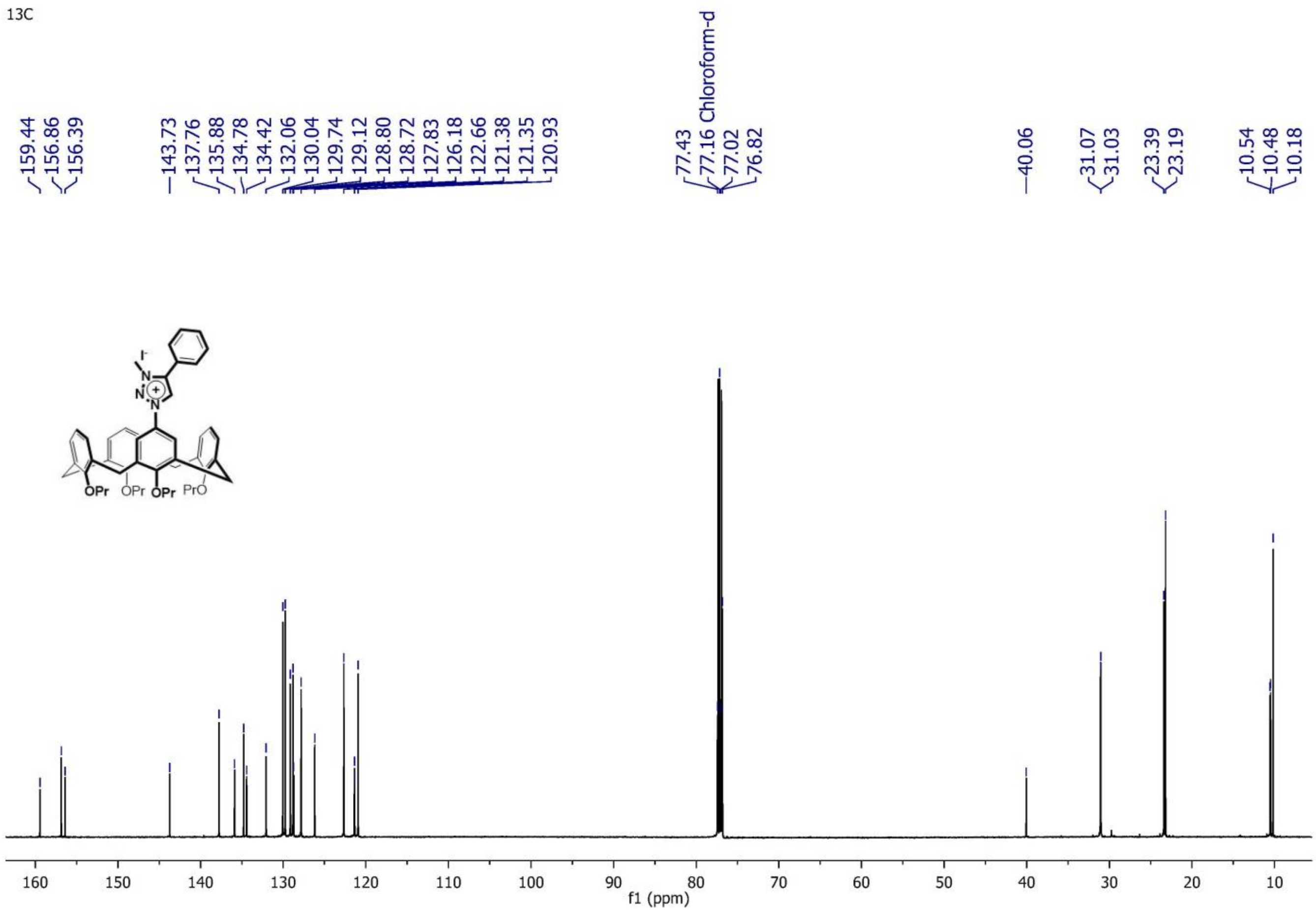

Figure S28: ^{13}C NMR spectrum of triazoliumcalix[4]arene (**12**)

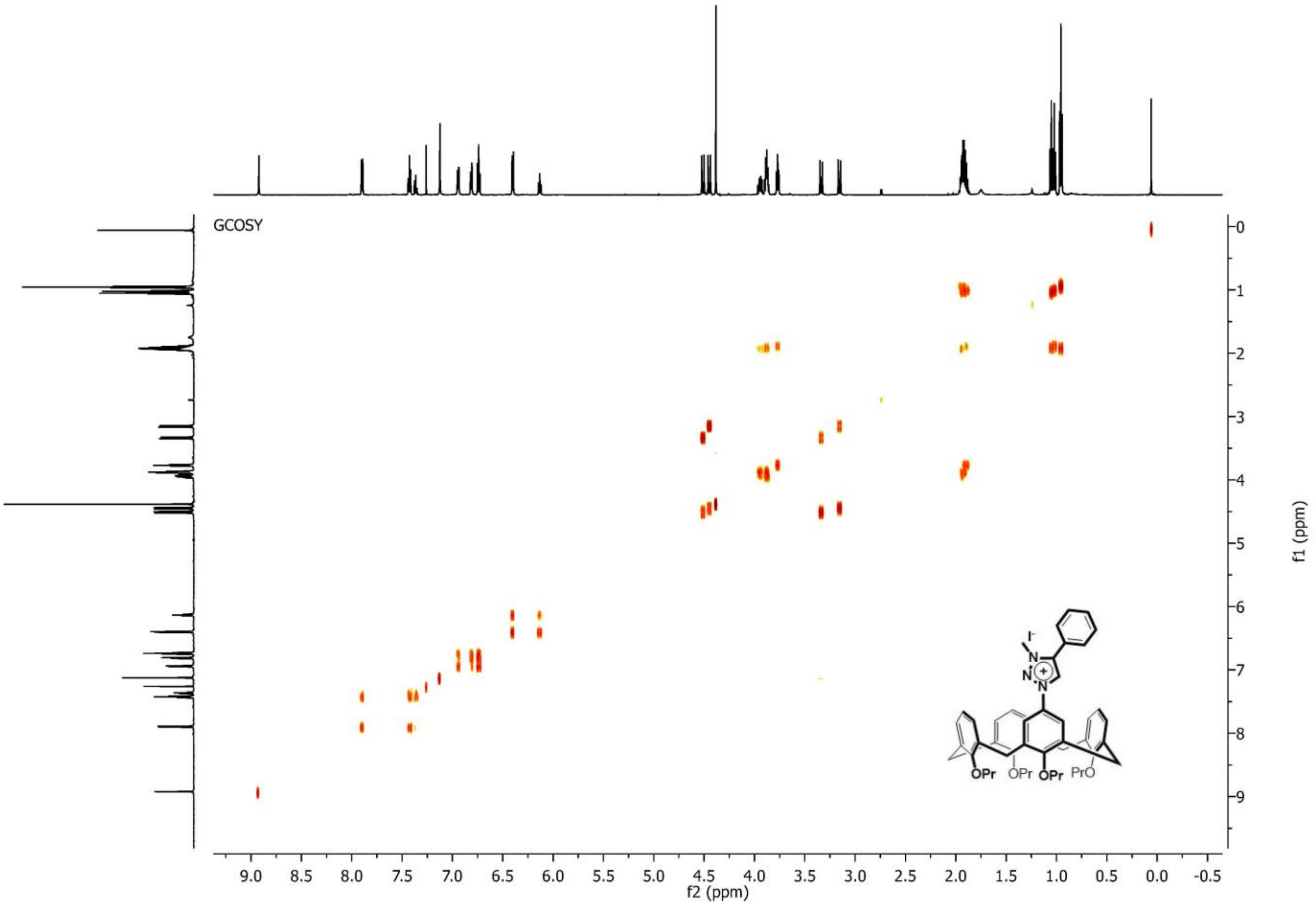

Figure S29: gCOSY spectrum of triazoliumcalix[4]arene (**12**)

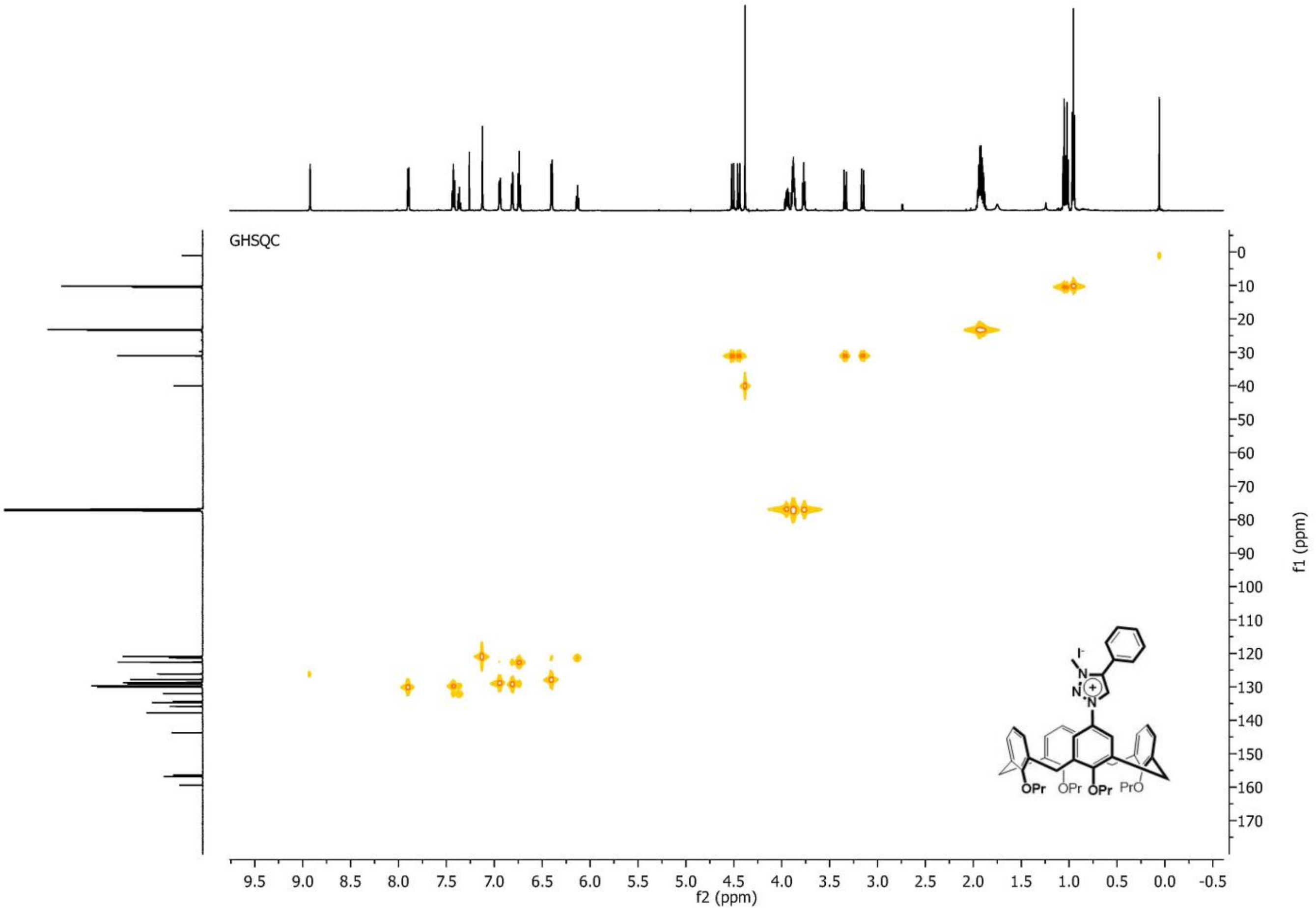

Figure S30: gHSQC spectrum of triazoliumcalix[4]arene (**12**)

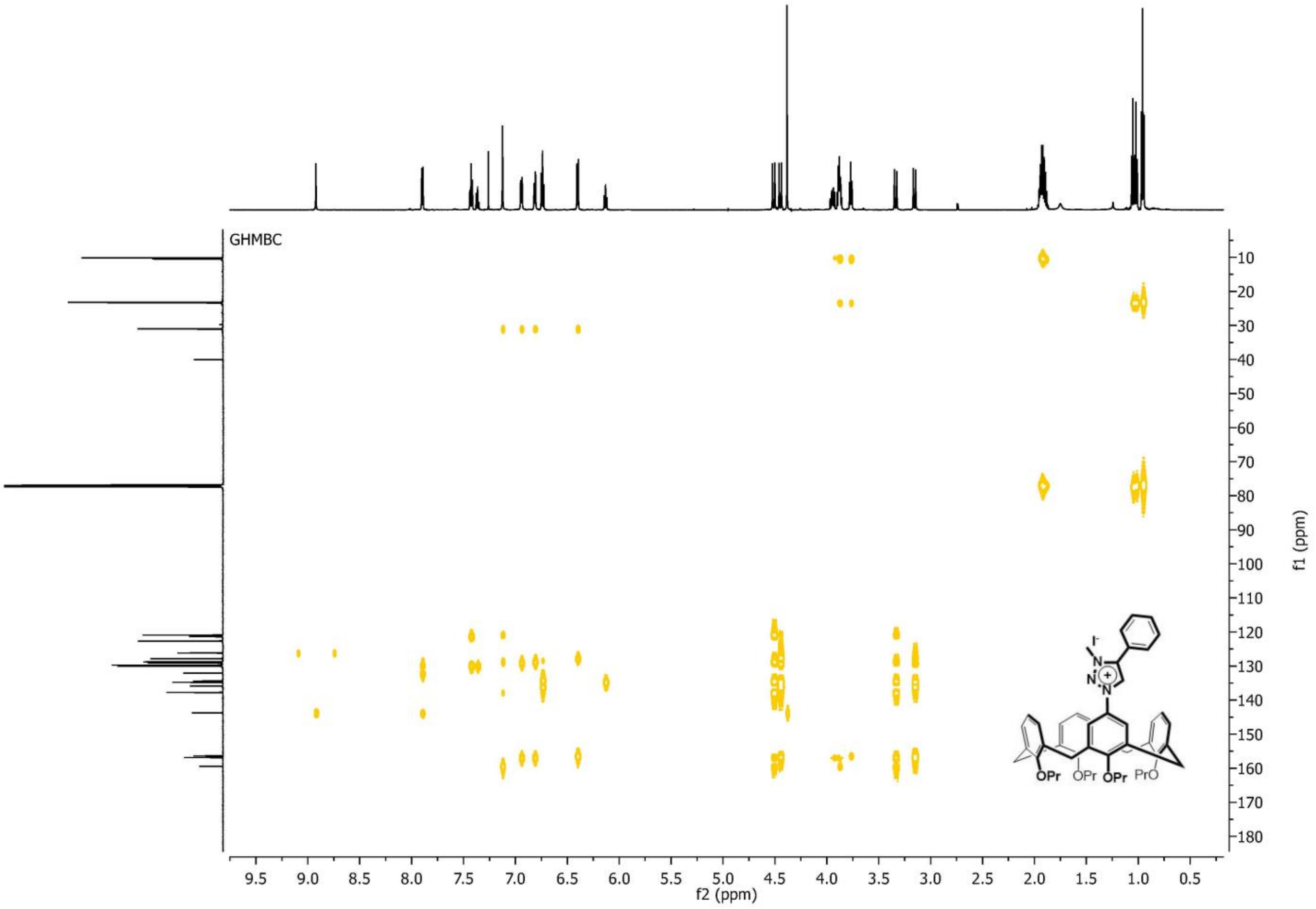

Figure S31: gHMBC spectrum of triazoliumcalix[4]arene (**12**)

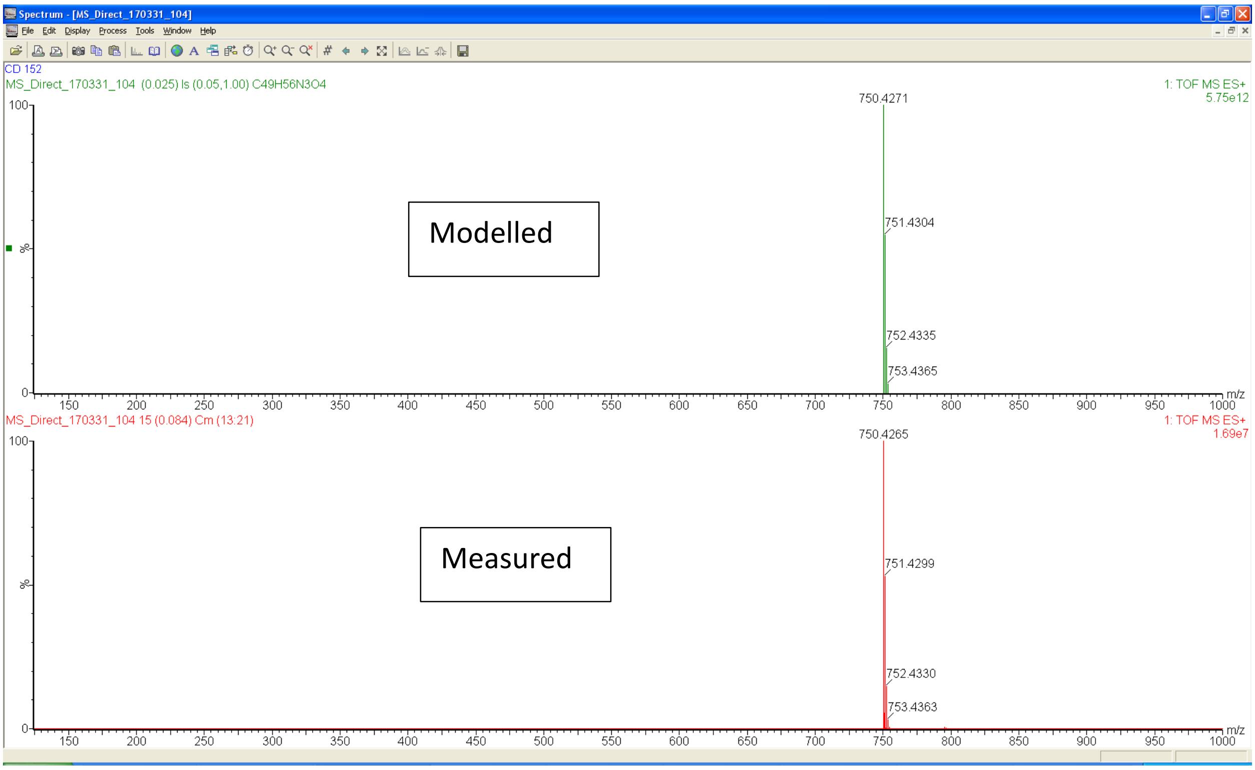

Figure S32: HRMS spectrum of triazoliumcalix[4]arene (**12**)

Attempted Ullman coupling to form calix[4]arene azide **7**.

Table S1: Ullmann-type coupling reaction conditions using acetone:H_2O.

Entry	Acetone/H_2O	DMEDA (equiv)	CuI (equiv)	NaN_3 (equiv)	Temp. (°C)	Time (h)	Yield (%)
**1**	8 mL:0.5 mL	0.15	0.1	2	85	24	mixture
**2**	4.8 mL:0.3 mL	0.15	0.1	2	85	24	mixture
**3**	8 mL:0.5 mL	0.3	0.2	4 + 2	85	26	mixture
**4**	10 mL:1 mL	0.3	0.2	2	90	24	mixture
**5**	10 mL:1 mL	0.3	0.2	2	80	48	mixture

Having successfully synthesised the model azide with both acetone and DMF, these solvents were then applied to the calixarene system. Acetone had provided the highest yield in the model reaction and was therefore tested first. The first calixarene reaction was performed using the same 7:3 (v/v) solvent ratio of acetone/H_2O as for the model reaction. The same equivalents of all reagents were added and the mixture was heated to 85 °C. Even at 85 °C the solubility of the starting material was poor and monobromocalix[4]arene was visible as a white solid floating around the reaction flask. The reaction was left to stir overnight, but TLC the next day indicated that no product had formed. It was postulated that there was too much water present in the reaction, which prevented the starting material from dissolving effectively. The hydrophobic nature of the starting material meant that the logical response was to repeat the reaction using less water. This was done by dissolving 200 mg of monobromocalix[4]arene **6** in 8 mL of acetone and 0.5 mL of distilled water, which resulted in a solvent ratio of 16:1 (entry 1, Table S1). The starting material dissolved instantly, which showed that a more dilute solution with less water was a step in the right direction in terms of solubility. The same equivalents of all reagents were added and the reaction mixture was degassed before being heated. At around 55 °C, the solution was dark turquoise in colour and became completely clear when heated to 85 °C. TLC analysis after 4 hours using 2% EtOAc/PET showed the formation of a single new spot which was slightly more polar than the starting material spot. A 10% solution of PPh_3 staining technique revealed that the lower spot was an azide. TLC indicated a substantial amount of remaining starting material, so the reaction was left to stir overnight. An almost identical result was obtained from TLC the next morning, and the reaction had unfortunately not gone to completion. The reaction was stopped after 24 hours regardless, by addition of 1 M HCl to the dark green solution. Purification via silica gel flash column chromatography turned out to be significantly more challenging than expected due to the similarity in R_f values of the starting material and product. Multiple attempts using different solvent systems all produced co-eluted fractions, which contained both starting material and product. FTIR analysis of the crude mixture produced an azide stretch at 2108 cm^{-1} which confirmed that the azide had formed, however, quantification had not yet been achieved. It was speculated that the reaction had been diluted too drastically when attempting to dissolve the starting material and this had inhibited reaction completion. The synthesis was repeated using the same 16:1 solvent ratio, reagent quantities and temperature as before (entry 2, Table S1). The concentration was increased by using 4.8 mL of acetone and 0.3 mL of distilled water however. TLC analysis after stirring under reflux for 24 hours at 85 °C indicated that a seemingly identical result to the previous reaction had been obtained.

The use of higher reagent quantities was next investigated. It was theorised that the hydrophobic cavity and sheer size of the calix[4]arene was preventing efficient contact of the reagents with the brominated active site. Table S1's third entry shows the conditions for the next attempted synthesis of monoazidocalix[4]arene **7**. Monobromocalix[4]arene **6** was added to 8 mL of acetone and 0.5 mL of distilled water, followed by addition of all reagents with doubled equivalents: NaN_3 (4 equiv), Na-ascorbate (0.1 equiv), CuI (0.2 equiv) and DMEDA (0.3 equiv). After the solvent was degassed, the mixture was stirred overnight under reflux at 85 °C. The reaction had not yet reached completion after 21 hours, so additional NaN_3 (2 equiv) was added and the reaction mixture was stirred for a further 5 hours. Unfortunately, after a total reaction time of 26 hours with increased reagent quantities, it was found by TLC that there was still starting material remaining. A change in TLC eluent from 2% EtOAc/PET to 35% DCM/PET brought to light that the previously suspected single azide spot was in fact three spots with extremely similar R_f values. Two of the three

spots were identified by co-spotting tetrapropoxycalix[4]arene **8** alongside the reaction mixture, and by using the previously described azide stain. Both the desired azide product and tetrapropoxycalix[4]arene **8** had formed, and were found to have essentially identical R_f values. It turned out that quenching of some kind had taken place during the reaction and formed the latter. The third compound, which was only marginally more polar than the two previously mentioned compounds, was not successfully identified. Solubility of NaN_3 had become a potential concern due to its insolubility in acetone and it was therefore decided to add more water to the next reactions. A solution of monobromocalix[4]arene **6**, NaN_3 (2 equiv), Na-ascorbate (0.1 equiv), CuI (0.2 equiv) and DMEDA (0.3 equiv) was prepared in acetone (10 mL) and distilled water (1 mL) (entry 4, Table S1). The reaction mixture was stirred under reflux at 90 °C for 24 hours after which TLC confirmed that a similar result to the previous reaction had been obtained. Although the reaction had not reached completion, a positive outcome was that the starting material spot seemed less prominent than before. The reaction was repeated one more time in the acetone/H_2O solvent system and stirred for 48 hours at 80 °C (entry 5, Table S1). This unfortunately did not lead to an improved result, so focus was shifted to a new solvent combination.

The Ullmann-type coupling reaction was next performed using a DMF/H_2O solvent system. Monobromo-calix[4]arene was dissolved in DMF (8 mL) and distilled water (1 mL). NaN_3 (2 equiv), Na-ascorbate (0.1 equiv), CuI (0.2 equiv) and DMEDA (0.3 equiv) were added to the flask and the mixture was heated to 100 °C after being degassed. The result after heating under reflux for 20 hours was a slightly cleaner reaction in which the unidentified compound from the previous reactions had not formed. The spot, which was thought to be the desired azide product, did not stain positively for an azide however, which did not bode well. It was considered that the copper catalyst was perhaps decomposing during the reaction. The synthesis was repeated using considerably higher reagent quantities in a more concentrated solution of DMF (5 mL) and distilled water (1 mL). NaN_3 (4 equiv), Na-ascorbate (0.2 equiv), CuI (0.4 equiv) and DMEDA (0.3 equiv) were added to the flask containing monobromocalix[4]arene **6** and the mixture was heated to 100 °C after being degassed. 18 hours of stirring under reflux did not produce a positive result. Predominantly monobromocalix[4]arene starting material and a negligible amount of the target azide were visualised using TLC.

Attempted nitration with acetic acid

Examples of tetrapropoxy-calix[4]arene mononitration found in the literature are based on a procedure reported by Reinhoudt and co-workers.[5] The procedure involved stirring tetrapropoxycalix[4]arene and glacial acetic acid (42 equiv) in DCM. After addition of 65% nitric acid (15 equiv), the mixture was stirred at room temperature for 30 minutes before being quenched with water. The product was extracted with DCM, after which the organic layer was washed with Na_2CO_3 and water before being dried over $MgSO_4$. Removal of the solvent in vacuo was followed by purification *via* silica gel flash column chromatography. From this, Reinhoudt and co-workers obtained a mixture of mononitro-calix[4]arene (30%) with traces of distal dinitro-calix[4]arene and proximal dinitro-calix[4]arene.

The first synthesis of mononitrocalix[4]arene **9** (entry 1, Table S2) was performed just as described by Reinhoudt and co-workers. After 30 minutes of stirring at room temperature (22 °C), the reaction was quenched by addition of water. Following the work-up, it was discovered that only starting material had been recovered. The reaction was repeated the next day using 70% nitric acid (entry 2, Table S2) and stirred at 23 °C. Slight darkening of the solution was observed, where before it had remained clear. The formation of a faint new spot near the baseline was observed from TLC and the reaction was quenched again after 30 minutes. A negligible amount of product had formed and it became apparent that a longer reaction time was necessary. Tetrapropoxycalix[4]arene was then stirred for 2 hours at room temperature (23 °C) in the presence of the two acids (entry 3, Table S2). After this time, TLC of the mixture showed the presence of starting material, a more prominent mononitrocalix[4]arene spot and a new spot which had formed near the baseline. After quenching with water, purification of the brown/orange crude mixture was achieved via silica gel flash column chromatography using an eluent gradient of 15% to 25% (DCM/PET). It was found that half of the tetrapropoxycalix[4]arene **8** starting material remained unreacted, and was obtained as the first compound. Mononitrocalix[4]arene **9** was obtained in the next fractions as a light yellow solid in only 21% yield. The column was then flushed to isolate the bottom spot, and a trace amount of what was presumed to be dinitrocalix[4]arene was obtained. The compound was not characterised, as the focus of this study was calix[4]arene mononitration.

The next attempt at improving the yield of the nitration reaction involved stirring tetrapropoxycalix[4]arene **8** in DCM with both acids at room temperature (20 °C) while monitoring the progress of the reaction every hour (entry 4, Table S2). After 7 hours of stirring, no product was visible and the solution remained clear. The reaction mixture was then left to stir overnight at room temperature. TLC the next morning of the pitch-black solution indicated that over-nitration had occurred throughout the night. No starting material was visible and only a trace amount of mononitrocalix[4]arene **9** could be observed. Both species had been converted to over-nitrated products, which had formed a prominent spot on the baseline. The TLC eluent's polarity was increased from 20% to 60% (DCM/PET) to move the spot off the baseline. Three compounds became visible, of which two had extremely similar polarities. It was theorised that these were both proximal- and distal dinitrocalix[4]arene, based on the findings of Reinhoudt and co-workers. The more polar spot was suspected to be trinitrocalix[4]arene. The synthesis was then repeated (entry 5, Table S2) while monitoring reaction progress via TLC every 30 minutes. As there was a distinct lack of control over the reaction in terms of over-nitration, a standardised method of TLC was performed to try and quantify formation of products by UV visualisation. A constant volume of 100 µL was removed from the reaction mixture every 30 minutes for 2.5 hours. These volumes were then diluted with a constant volume of DCM (0.1 mL) and spotted twice on the silica TLC plate using the same spotter. The idea was to quench the reaction when it seemed like the rate of mononitrocalix[4]arene **9** formation had decreased and the rate of over-nitrated product formation had increased. It was a shot in the dark as there was no way of knowing the relationship between these two rates. It was also not possible to know whether over-nitration was occurring on the starting material, mononitrocalix[4]arene or both. After the first hour of stirring at 20 °C, the colour of the solution had become much darker but no product was visible from TLC. In the next hour, a faint product spot had formed. The spot had become noticeably darker within the next 30 minutes, but the formation of a by-product which was only slightly more polar than the desired compound was also noted. The reaction was stirred for a further 15 minutes before being quenched, which marked a total reaction time of 2 hours and 45 minutes. At this point, the by-product spot had become considerably darker and spots of over-nitrated products had started to form on the baseline. Formation of a spot, which was extremely close to the product spot, meant that co-elution was a concern when purifying the crude mixture. For this reason, the volume of silica gel was increased when flash column chromatography was performed. Mononitrocalix[4]arene **9** was obtained as pure compound in 28% yield, with just more than a third of the starting material which remained unreacted. This was an improved result in terms of yield and quantity of unreacted starting material.

Table S2: Mononitration of tetrapropoxycalix[4]arene **8** using HNO_3 and HOAc.

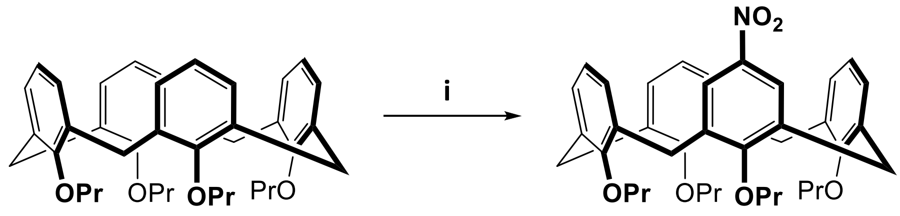

8 9

Reagents and conditions: i) As per table with HNO_3 (15 equiv), HOAc (42 equiv), DCM.

Entry	Temperature (°C)	Time	Mononitro yield (%)
**1****	22	30m	–
**2**	23	30m	–
**3**	23	2h	21
**4**	20	overnight	over-nitration
**5**	20	2h 45m	28
**6**	20 to 23	2h 50m	44
**7**	23 to 30	1h	–
**8**	20	1h	–
**9**	20	3h	–

The synthesis was then repeated in a similar fashion (entry 6, Table S2). TLC was only performed after 1.5 hours, as not much product had formed up until this point in the previous synthesis. The solution had become pitch-black in colour after 2 hours and 50 minutes of stirring, and the same by-products were visible from TLC. The reaction was quenched for fear of losing calix[4]arene **9** to over-nitrated products. It was noted that the room temperature had increased from 20 °C to 23 °C throughout the reaction. After purification via silica gel flash column chromatography it was discovered that less than a third of the starting material remained unreacted and mononitro-calix[4]arene **9** had formed in 44% yield. This was a clear improvement in terms of yield and unreacted starting material. It was theorised that a temperature increase of merely 3 °C throughout the reaction had led to a significant increase in yield, even though the reaction time was only 5 minutes longer.

At this point it had become clear that darkening of the solution was an indication that nitration had started to occur. Stirring of the next reaction was started at a room temperature of 23 °C (entry 7, Table S2). The solution remained clear after 35 minutes of stirring, so the reaction was heated to 30 °C and stirred for a further 25 minutes. No colour change was observed, so the reaction was quenched with water and the starting material extracted with DCM. This result was compared to entry 4 of Table S2, of which the solution remained clear for 7 hours. Over-nitration had then occurred when the reaction was left to stir overnight. It seemed as if the nature of the nitration was in some way autocatalytic, and that the reaction would not proceed if the initial nitration did not occur. It was decided to repeat the reaction with the extracted starting material. If a colour change was not observed after 1 hour of stirring, the reaction would be quenched. The next two reactions (entries 8 and 9, Table S2) provided nothing but frustration. After stirring for 1 and 3 hours respectively, no colour change was observed for either reaction. The inconsistency of this

** It should be noted that 65% nitric acid was used for this reaction. The remaining entries all used 70% nitric acid.

reaction would not suffice when generating mononitro-calix[4]arene **9** material for use in subsequent reactions. A new nitration method was therefore investigated.

Attempted formation of the ruthenacycle **13**

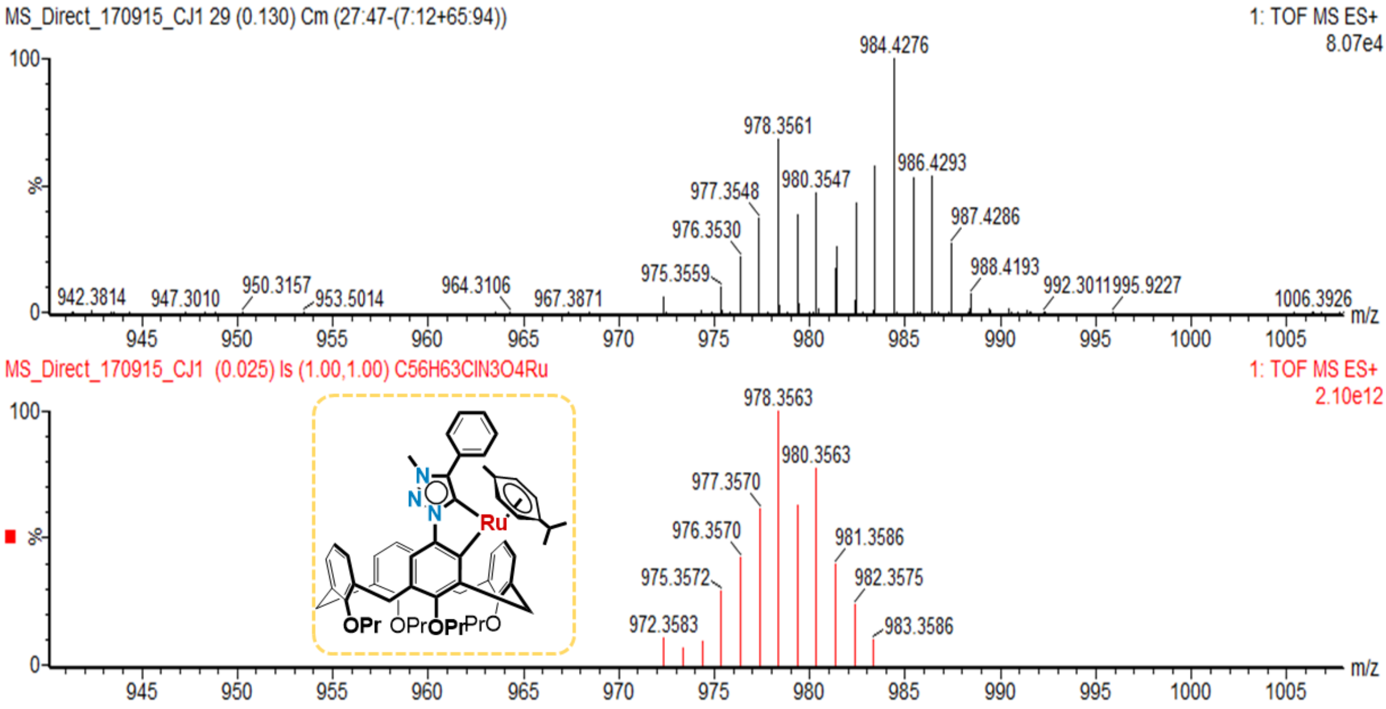

Figure S33. Zoomed in section of the HRMS spectrum showing the predicted (bottom) and acquired (top) section that corresponds to the ruthenacycle.

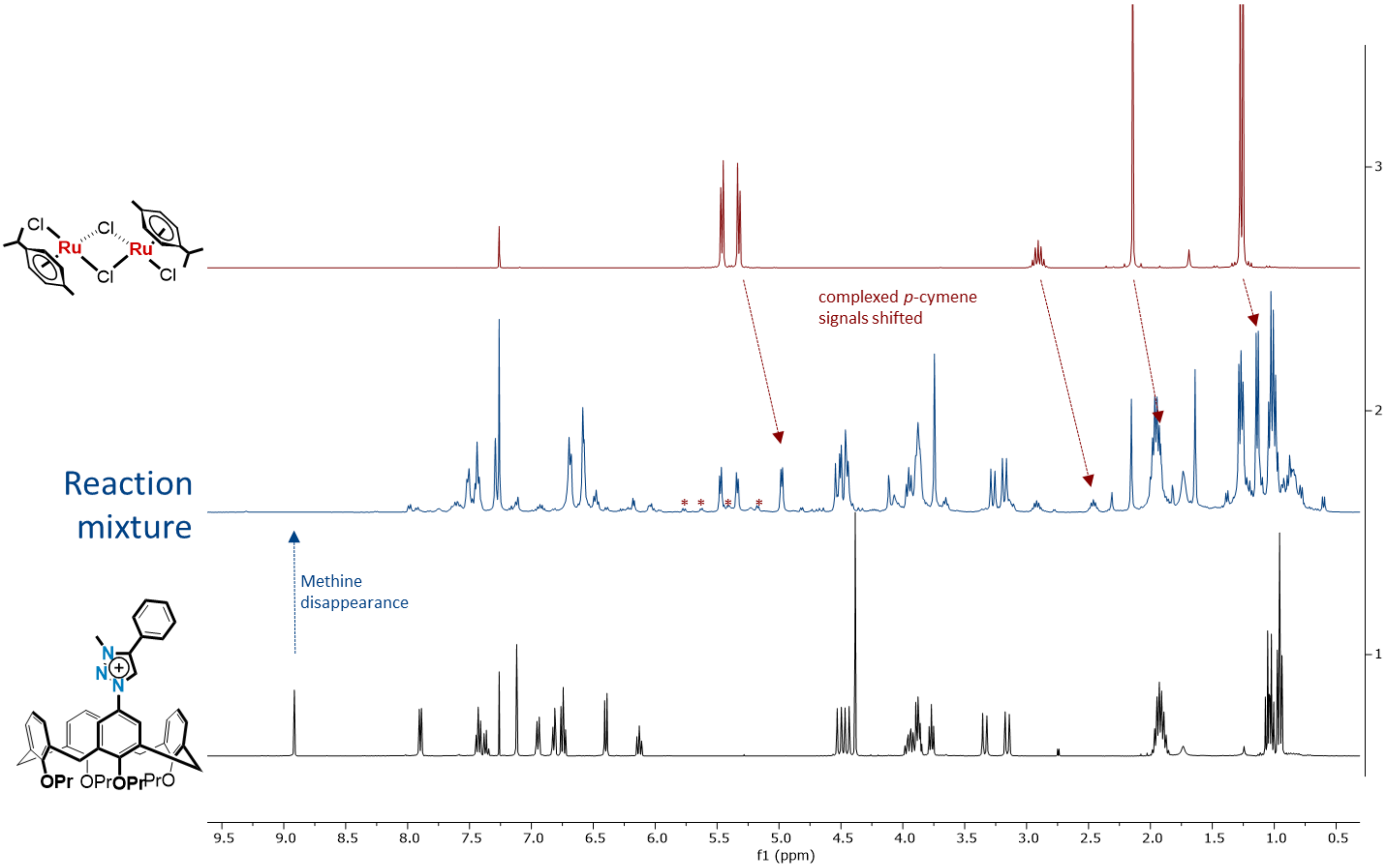

Figure S34. Stacked plot of NMR spectra showing the ruthenium dimer (top), reaction mixture (middle) and starting material (bottom). Important points are: 1: disappearance of the triazole methine indicating C–M bond formation; 2: shifts in the *p*-cymene signals consistent with complex formation; and 3: signals (*) that one might expect for diastereomers when *p*-cymene is part of a ruthenacycle (speculative, but suggests very low yield).

References

(1) Burg, A. B.; Liebman, J. F.; Greenberg, A. *Purification of Laboratory Chemicals*, 3rd ed.; Oxford, 1988; Vol. i.

(2) Bang, J. S.; Kim, Y. J.; Song, J.; Yoo, J. S.; Lee, S.; Lee, M. J.; Min, H.; Hwang, K. W.; Min, K. H. *Bioorganic Med. Chem.* **2012**, *20* (17), 5262–5268.

(3) Bennett, M. A.; Huang, T.-N.; Matheson, T. W.; Smith, A. K.; Ittel, S.; Nickerson, W. *Inorg. Synth.* **1982**, 74–78.

(4) Dondoni, A.; Marra, A.; Scherrmann, M.-C.; Casnati, A.; Sansone, F.; Ungaro, R. *Chem. - A Eur. J.* **1997**, *3* (11), 1774–1782.

(5) Kelderman, E., Derhaeg, L., Heesink, G. J. T., Verboom, W., Engbersen, J. F. J., van Hulst, N. F., Persoons, A., Reinhoudt, D. N. *Angew. Chem. Int. Ed. Engl.* **1992**, *31* (8), 1075–1077.

(6) Van Wageningen, A. M. A.; Snip, E.; Verboom, W.; Reinhoudt, D. N.; Boerrigter, H. *Liebigs Annalen*. 1997, pp 2235–2245.
